# PTEN Regulates Microtubule Polymerization via mTORC2, But Not mTORC1, in Peripheral Sensory Neurons

**DOI:** 10.1523/ENEURO.0116-26.2026

**Published:** 2026-09-08

**Authors:** Simone Evans, Julia Reglewski, Haley Rose, Cara Lewis, Anika Nayak, Cole Minsky, Evan McNeil, Brizha Knowles, Wei Wang, Meijie Li, Bryan W. Luikart, Jennifer Hong

**Affiliations:** ^1^Department of Integrative Neuroscience, Guarini School of Graduate and Advanced Studies, Dartmouth College, Hanover, New Hampshire 03755; ^2^Geisel School of Medicine at Dartmouth, Hanover, New Hampshire 03755; ^3^Department of Neurosurgery, Beth Israel Deaconess Medical Center, Boston, Massachusetts 02215; ^4^Department of Molecular and Systems Biology, Geisel School of Medicine, Dartmouth College, Hanover, New Hampshire 03755; ^5^Department of Neurobiology, Heersink School of Medicine, University of Alabama at Birmingham, Birmingham, Alabama 35233; ^6^Departments of Surgery and Molecular and Systems Biology, Geisel School of Medicine, Dartmouth College, Hanover, New Hampshire 03755

**Keywords:** axon, cytoskeleton, peripheral regeneration, PTEN, tubulin

## Abstract

Peripheral neuropathy affects over 18 million adults in the United States ( Hicks et al., 2021), but therapeutic outcomes are poor due to a lack of regenerative treatments. Development of novel, disease-modifying therapies is hindered by poor understanding of the mechanisms promoting axonal regeneration in peripheral neurons. Pten is a strong negative regulator of cell growth, and Pten-KO drives axonal regeneration in various neuronal subtypes potentially via downstream regulation of stability of the microtubule (MT) cytoskeleton, a vital component of axonal growth. While Pten-KO accelerates MT polymerization rates in the axonal growth cone, it remains unknown whether this action is dependent on mTORC1 or mTORC2 signaling and regeneration under Pten-KO is dependent on MT activation. Here, we perform in vitro codeletions of either Raptor (mTORC1) or Rictor (mTORC2) alongside Pten-KO in mouse peripheral sensory neuron cultures of either sex to isolate the effects of each pathway on the MT cytoskeleton. We use Pten-KO to increase MT polymerization and neuronal outgrowth and then show that suppression of mTORC2, but not mTORC1, is sufficient to reduce the accelerated MT polymerization and neuronal hypertrophy to wild-type levels. These results are specific to the axonal growth cone, and MT dynamics in the proximal axon shaft are not impacted by Pten-KO, mTORC1 suppression, or mTORC2 suppression. Our results help elucidate the mechanism by which Pten regulates the MT cytoskeleton and axonal outgrowth in peripheral sensory neurons, localize where this occurs in the axon, and highlight the MT cytoskeleton as a potential molecular target for regenerative therapies.

## Significance Statement

Pten-KO promotes neuron growth, but Pten has several downstream effectors, and it is unclear which effectors drive axonal regeneration. One of these downstream targets is the microtubule cytoskeleton, which is vital for normal axonal growth and development. We demonstrate that Pten-KO increases MT polymerization rates in the growth cone, but not proximal shaft, in axons of peripheral sensory neurons. This effect is reversed by codeletion of mTORC2, but not mTORC1, and the loss of mTORC2 primarily inhibits elongation of the distal axon, suggesting that the regulation of MT polymerization is specific to the growth cone. These results highlight MT dynamics in the axonal growth cone as a vital component of peripheral neuron regeneration and a potential therapeutic target.

## Introduction

Peripheral neuropathy affects over 18 million people in the United States, with 200,000 traumatic nerve injuries undergoing nerve repair procedures in the United States each year (Ichihara et al., 2008). An additional 30% of chemotherapy recipients (Seretny et al., 2014) and at least 50% of Type II diabetes patients (Feldman et al., 2019) will also develop chronic peripheral neuropathy. The currently available treatments for peripheral neuropathies are limited and only address symptoms of nerve damage without modifying disease progression or restoring nerve function (Feldman et al., 2019; Burgess et al., 2021). There is a critical need for novel, regenerative therapies, but a lack of understanding of the molecular pathways regulating peripheral nerve regeneration prevents development of more effective treatments.

Pten is a widely acting negative regulator of cell growth, and Pten-KO has been shown to promote neurite outgrowth in a variety of neuronal subtypes (Park et al., 2008; Christie et al., 2010; Liu et al., 2010; Kwon et al., 2001; Karalis et al., 2022; Elmalky et al., 2024). However, directly targeting Pten for regenerative therapies is challenging, due to its extremely broad downstream action and strong oncogenic potential. Not only does loss of Pten in almost any cell type lead to cancer in animal models (Suzuki et al., 1998; Chalhoub and Baker, 2009), but human Pten mutations are linked to significantly increased lifetime risk of cancer (Hollander et al., 2011; Yehia et al., 2023). Therefore, leveraging Pten-related growth pathways to promote nerve healing will require a better understanding of its molecular mechanisms to find targeted therapies.

Two major signaling targets of Pten are the complexes mTORC1 and mTORC2. Understanding how these complexes affect neuronal growth and regeneration may provide alternative, more selective targets for therapeutic intervention that avoid tumorogenesis concerns that result from general Pten-KO. One potential effector of Pten-KO-mediated growth is the microtubule cytoskeleton. Microtubules are polar, self-assembling polymers, composed of a coiled chain of alternating alpha- and beta-tubulin monomers. Microtubules polymerize and depolymerize from the “plus” end, which has an exposed beta-tubulin subunit and serves as an important binding site for regulatory proteins, such as end-binding protein 3 (EB3). Previous work has shown that Pten-KO in hippocampal neurons have accelerated MT polymerization in vitro (Getz et al., 2022), and other studies have found that Pten may impact MT stability (Kath et al., 2018). MTs are critical for axon growth and guidance, particularly in the growth cone (Y. Liu et al., 2015; Sánchez-Huertas and Herrera, 2021) and therefore comprise a promising molecular target for regenerative therapies. However, the impact of modulation of MT dynamics on peripheral nerve regeneration and repair has not been well studied, nor is it clear how Pten regulates these dynamics.

Here, we investigate PTEN-mediated regulation of MT dynamics by visualizing MT polymerization in cultured primary dorsal root ganglion neurons, using fluorescently labeled EB3 (EB3-GFP) to quantify movement of MT plus-ends in live cells. We hypothesize that PTEN drives changes in neuronal morphology by modulating MT dynamics. Next, we use PTEN-KO to drive neuronal outgrowth and then perform simultaneous in vitro knockouts of either Raptor (a necessary component of mTORC1) or Rictor (a necessary component of mTORC2), both downstream of Pten, to identify their potential impact on the cytoskeleton and cellular growth. We propose a mechanistic pathway between PTEN and MT polymerization in the axonal growth cone and observe that PTEN differentially regulates MT polymerization across subcellular compartments in sensory neurons. Finally, we show the relationship between MT dynamics and overall neuronal morphology and highlight the MT cytoskeleton as a vital component of peripheral nerve regeneration that may have future clinical applications.

## Materials and Methods

### Animals

All animal procedures were performed in accordance with the Geisel School of Medicine at Dartmouth’s animal care and use committee’s regulations. Both sexes were used for all experiments.

*Pten^fl/fl^* × *Raptor^fl/fl^* and *Pten^fl/fl^* × *Rictor^fl/fl^* animals were generated by the Hong lab at Geisel School of Medicine at Dartmouth as follows. *Pten^fl/fl^* animals (B6.129S4-Ptentm1Hwu/J with C57BL/6J background from Jackson Laboratory) were crossed with *Raptor^fl/fl^* animals (Jackson Labs stock: 013188) to generate *Pten^fl/fl^* × *Raptor^fl/fl^* animals. *Pten^fl/fl^* animals (B6.129S4-Ptentm1Hwu/J with C57BL/6J background from Jackson Laboratory) were crossed with *Rictor^fl/fl^* animals (Jackson Labs stock: 020649) to generate *Pten^fl/fl^* × *Rictor^fl/fl^* animals. Animals were kept on a 12 h light/dark cycle with *ad libitum* access to chow and water. Animals were killed at age >90 d.

### Primary neuronal cultures

#### Plate and media preparation

Plates were prepared by coating with 1 mg/ml poly-ʟ-lysine (Sigma-Aldrich, P4707) in borate buffer and incubated overnight at 37°C. On the day of culturing, plates were rinsed three times with sterile water and then preincubated with culture media. Culture media was made by combining 100 μl of 200 mM ʟ-glutamine, 400 μl of 50× B-27 (Thermo Fisher Scientific/Invitrogen, 17504044) supplement, 200 μl of 100× penicillin/streptomycin, and Neurobasal-A (Thermo Fisher Scientific/Invitrogen, 10888022) to 20 ml and then filtering the solution through a 0.22 μm filter.

#### Dissection

Mice were anesthetized by intraperitoneal injection of 0.6–0.8 ml 2% Avertin (2,2,2-tribromoethanol; Sigma-Aldrich, T48402). Scissors were used to make an incision into the ventral abdomen, puncture the diaphragm, and then cut anteriorly through the ribcage. Death was confirmed via cardiac puncture and then by cutting through the cervical spine. The spine was then isolated and the spinal cord extruded using an 18 g needle and saline. The spinal canal was bisected and spinal sections were placed into a dissecting dish of ice-cold HBSS (Hanks balanced salt solution, Sigma-Aldrich H2387), and DRGs were dissected out of the neural foramen under a stereoscope using forceps. Nerve roots and the epineurium were removed and dissected DRGs were placed into ice-cold prep medium, in a 15 ml tube. Prep medium was made fresh each culture set and was made from 140 μl of 1 M HEPES (4-(2-hydroxyethyl)-1-piperazineethanesulfonic acid, Thermo Fisher Scientific/Invitrogen, 15630080), 200 μl of 30% glucose stock solution (in sterile water), 100 μl of 100× penicillin/streptomycin, and 1× MEM (Thermo Fisher Scientific/Invitrogen, 11140050) to 10 ml.

#### Dissociation and purification

Isolated DRGs were allowed to settle, while collagenase-II solution was prepared from 10 mg collagenase-II (Sigma-Aldrich, C2-BIOC) in 1 ml of dissociation solution. Dissociation solution was prepared from 350 μl of 1 M HEPES, 500 μl of 100× penicillin/streptomycin, and 50 ml of HBSS. Collagenase solution was warmed for 10 min. Supernatant was removed from the tube of DRGs, and the DRGs were rinsed three times with cooled dissociation solution, each time with supernatant discarded. After the last rinse, 2 ml of dissociation solution was left in with the DRGs and 1 ml of warmed collagenase solution added. DRGs were incubated 1 h at 37°C, 5.0% CO_2_, with the tube gently agitated every 10 min. After 40 min (of the 1 h total incubation), a 150 μl of aliquot of 2% trypsin solution was removed from the −20°C freezer and warmed at 37°C. Then 20 min later (1 h total DRG incubation), the full 150 μl of aliquot was added to the DRGs in solution, gently agitating. Solution was then incubated at 37°C, 5.0% CO_2_ for an additional 9 min, agitating every 3 min. After incubation, supernatant was removed, and DRGs were rinsed once with 5 ml of cooled dissociation solution. DRGs were allowed to settle and supernatant was removed to 1.5 ml. A prepolished silanized glass pipette (Thermo Fisher Scientific, BrainBits, NC0319875) was used to triturate the DRGs until solution was homogenous. After homogenizing, solution was centrifuged for 10 min at 160 × *g* with soft stop at room temp. During centrifugation, 10 ml solution of 10% v/v DHS (Cytiva HyClone, donor equine serum, SH30074.04) in cell culture media (warmed to 37°C) was prepared. After centrifugation, supernatant was discarded and pellet rehomogenized using fire-polished pipette in 2 ml of the 10% DHS/media. This was centrifuged again for 10 min at 160 × *g* with soft stop. Supernatant was discarded and pellet rehomogenized using fire-polished pipette in 37°C cell culture media without DHS.

#### Plating

Media was removed from prewarmed, poly-ʟ-lysine-prepared culture plates (as previously described in Plate and media preparation). Solution of rehomogenized cells in culture media was dropped into wells in the center of culture plates, ∼0.5 ml per plate. Plates were kept for 2 h at 37°C, 5.0% CO_2_, and then gently flooded with prewarmed 10% DHS/media (v/v). After 24 h media was aspirated and replaced with media + 10% DHS (v/v) + beta-NGF + 80 μM 5fdu + 80 μM uridine.

#### Culture maintenance

Every 48 h, culture media was gently aspirated and replaced with prewarmed supplemented (10% v/v DHS + 5fdU + Uridine + beta-NGF) media.

#### Transfection

At DIV3, the cultures were cotransfected with two plasmids using calcium phosphate precipitation (Kwon and Firestein, 2013). The two plasmids used were EB3-GFP, to label fluorescently label MT plus-ends, and either a control mCherry plasmid (RedRubi) or a mCherry-Cre recombinase plasmid (pRubi-C-T2A-Cre). This yielded a dual transfection efficiency of ∼10%, allowing for imaging of isolated neurons in sparsely labeled cultures.

#### Vinblastine treatment

At DIV6–7, immediately before live imaging, 2 nM of vinblastine treatment were administered by adding vinblastine stock solution directly to the culture dish. All cells on that dish were then imaged 30–120 min after adding the vinblastine. A 1 mM vinblastine stock solution was prepared in MEM, and a 10 μM working solution was made fresh in PBS for each set of cultures.

#### Live imaging

At DIV6–7, the cultures were at live-imaged at 37°C, 5.0% CO_2_ incubation, using a spinning-disk microscope (Nikon Ti-E inverted). Transfected neurons were identified by screening for RFP+ cells, then taking 120 consecutive frames at ∼0.79 fps. All cells were imaged at 60× and at two *z*-slices, with the exact interval varying by image but usually within 1–5 μm.

#### Immunocytochemistry

Within 24 h of live imaging, cultures were immunostained. Briefly, cells were fixed with 50% (in existing plate media) of 4% PFA for 10 min and then 100% PFA for 10 min. Plates were rinsed with PBS, permeabilized using 0.4% Triton X-100 in PBS for 10 min, and then rinsed with PBS again. For plates stained for Pten, antigen retrieval was performed. For all other stains, plates were blocked using 10% DHS in PBS for 30 min and then incubated with primary antibody in 2.5% DHS in PBS overnight at 4°C. Plates were rinsed with PBS and then incubated with secondary antibody in 2.0% DHS in PBS overnight at 4°C. Plates then received a final PBS rinse and were mounted with Vectashield Antifade Mounting Medium (Vector Labs, H-1000-10) and sealed. Images were taken within 2 weeks of fixation on an LSM-520 confocal microscope (Zeiss). Primary antibodies used were anti-Beta-III-tubulin (Abcam, ab18207), anti-RFP (Rockland 600-901-379), and anti-Pten (Cell Signaling, 9559). Secondary antibodies used were supplied by Jackson Immunolabs.

#### Confocal imaging and morphological quantification

Fixed cells were imaged using a Zeiss LSM880 confocal microscope using a 20× objective. For morphology analysis, Z-stacks were taken with 1 μm step. Soma size was quantified using ImageJ at the *z*-step with the maximum soma area. Neurite quantification was performed by reconstructing the neurons using 3D, user-guided tracing in the software Neurolucida and then performing Sholl analysis in the software Neurolucida explorer. Briefly, a digital, 3D representation of the neurites was created, and Sholl analysis was performed by drawing concentric circles with 10-μm-radii intervals. Neurolucida Explorer calculated and reported the length of the radii between two adjacent circles and the number of times a circle intersected with a radii. Total neurite length was calculated by summing all Sholl of length (i.e., the length of all neurites within a window of radii, for all radii) for each cell.

#### Statistical analysis

All statistical analyses and graphical representations of the data were generated in GraphPad Prism 10, unless otherwise noted. For all microtubule data, all microtubule velocities across all timepoints for a single cell were averaged to generate a single data point, and reported *n* values are the number of cells. Reported mean and standard deviation are the mean and standard deviation across all cells per condition. For all morphological (soma size, Sholl analysis) data, *n* values are a single cell. For both microtubule and morphological experiments, data from at least three distinct culture sets (in other words, three individual animals) was reported for each genetic condition, to compensate for potential individual variability. Minimum sample size was determined by performing a power analysis based on prior work from our lab (citations removed for anonymity).

Exact statistical tests used are reported in relevant figure legends. We used Welch’s *t* test to compare manual versus automated tracking and did not perform multiple comparisons (Fig. 1). To compare MT velocities, soma size, sum of intersections, and sum of length across genetic conditions, we first used Welch and Brown–Forsythe one-way ANOVA tests to determine significance and then used Dunnett’s T3 multiple-comparisons test to examine specific intercondition comparisons (Figs. 2–4; Extended Data Figs. 4-1–4-6). To compare Sholl analysis results across genetic conditions, we performed a two-way ANOVA, with radius and condition as factors. We then used Tukey’s multiple-comparisons tests to examine differences across genetic conditions (Figs. 2, 3).

**Figure 1.**
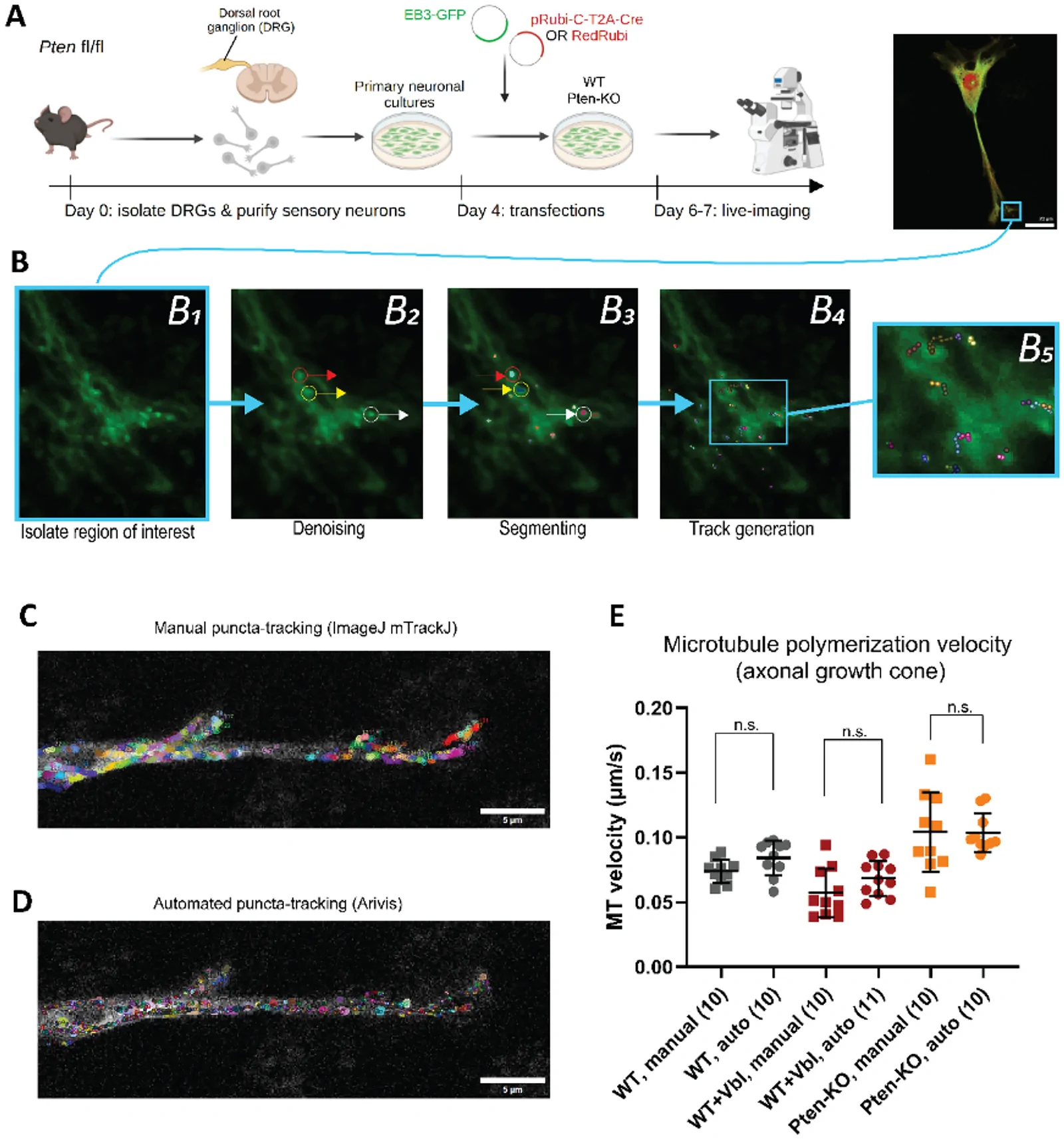
Using a system of primary neuronal cultures and an automated puncta-tracking pipeline to visualize and quantify microtubule polymerization in live cells. ***A***, Experimental design. Sensory neurons from DRGs were isolated from adult animals and cultured. Neurons were transfected on DIV3–4 with an EB3-GFP plasmid, to label MT plus-ends, and either a RedRubi plasmid (control) or pRubi-C-T2A-Cre plasmid (generates knockouts). Neurons are live-imaged on DIV6–7 (120 frames, 0.79 fps). ***B***, Representative still images obtained of EB3-GFP puncta in the axon growth cone from development of automated EB3-GFP puncta-tracking pipeline demonstrating preprocessing steps of (***B_1_***) enlarging region of interest, (***B_2_***) denoising, and (***B_3_***) segmenting puncta followed by (***B_4_***) track generation and quantification of overall track lengths shown in magnified view (***B_5_***). ***C***, Representative still image of manual (ImageJ mTrackJ) puncta-tracking in an axonal growth cone, showing all tracks across the entire movie. ***D***, Representative still image of automated (arivis) puncta-tracking in an axonal growth cone, showing all tracks across the entire movie. ***E***, No significant difference between manual and automated puncta-tracking for the same set of 10 WT cells (Welch’s *t* test, *p* = 0.0585), 10 vinblastine-treated cells (Welch’s *t* test, *p* = 0.1366), or 10 Pten-KO cells (Welch’s *t* test, *p* = 0.9631). Overall, manual puncta-tracking produces fewer (*n* tracks = 114.9 ± 70.77), but longer (track length = 0.6128 ± 0.1576 μm), MT tracks, while automated tracking produces more (*n* tracks = 336.3 ± 265.3), shorter (track length = 0.2435 ± 0.07774 μm) tracks (Extended Data Fig. 1-1). Created with BioRender.com.

10.1523/ENEURO.0116-26.2026.f1-1 Figure 1-1 *Additional comparisons of manual and automated puncta-tracking*. A. Comparisons of track length between manual and automated tracking for 10 imagesets (cells). Manual tracking produces significantly longer tracks (Welch’s t-test, p < 0.0001). B. Comparisons of number of tracks produced between manual and automated tracking for 10 imagesets (cells). Automated tracking produces significantly more tracks (Welch’s t-test, p = 0.0283). Download Figure 1-1, TIF file.

**Figure 2.**
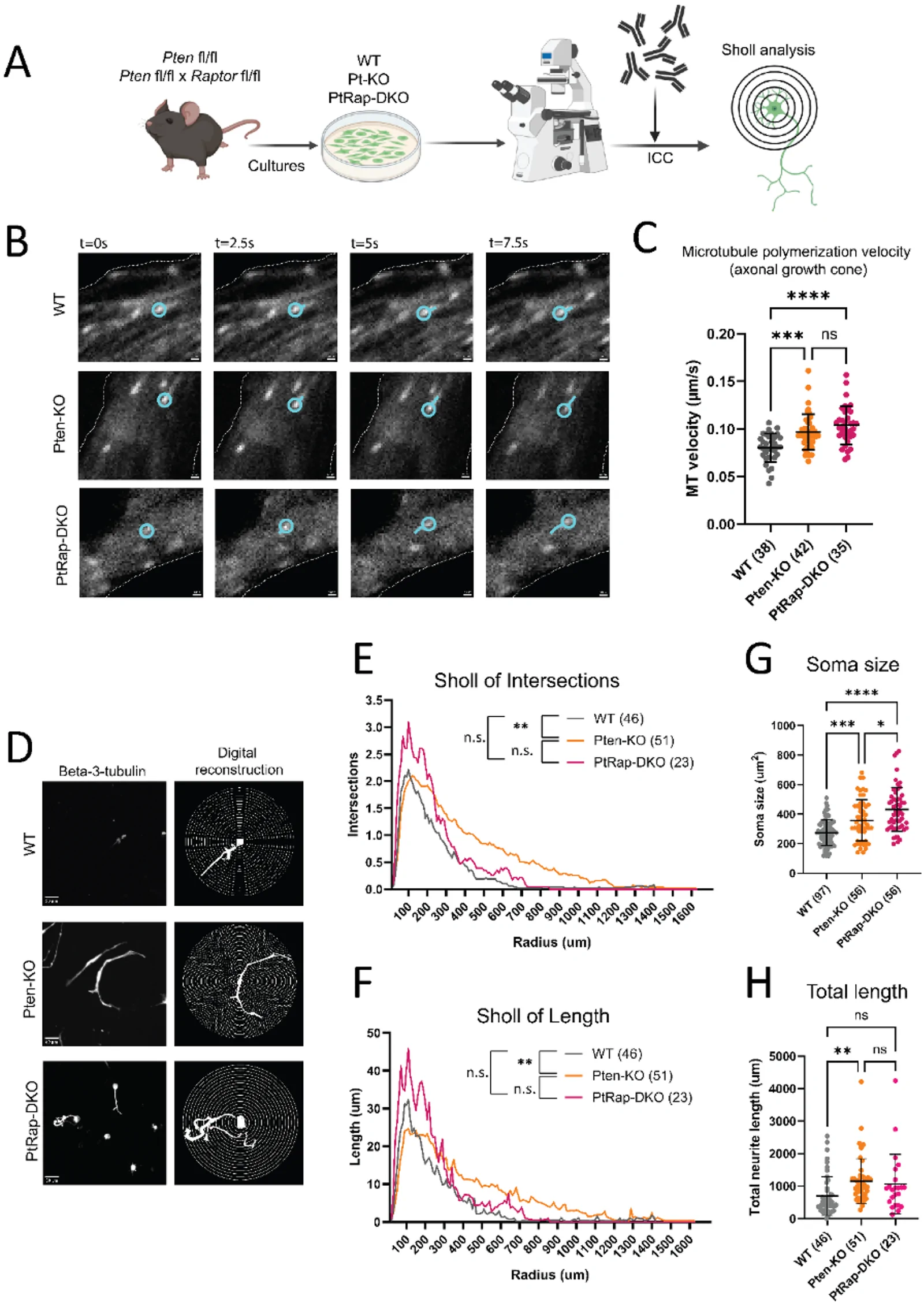
Genetic disruption of mTORC1 does not inhibit Pten-KO-mediated axon growth in vitro. Codeletion of *Pten* and *Raptor* in cultured DRG neurons fails to inhibit *Pten*-KO-mediated increase in MT polymerization velocities. Immunostaining for Beta-III-tubulin reveals increase axon outgrowth compared with WT neurons by quantitative Sholl analysis. ***A***, Experimental design. Dorsal root ganglia are harvested fresh from adult *Pten*^fl/fl^ animals or adult *Pten*^fl/fl^ *Raptor*^fl/fl^ animals and primary sensory neurons are then dissociated and grown in primary culture. At day in vitro (DIV) 4, the neurons are transfected with a plasmid encoding EB3-GFP to label plus-end microtubules and Cre recombinase (pRubi-C-T2A-Cre) to conditionally delete *Pten* and *Raptor* or EB3-GFP and RFP (RedRubi) as negative controls (*Pten* WT). At DIV 6-7 the cultures are live-imaged to generate movies of EB3-GFP puncta movement. After imaging, the cells are fixed and subsequent immunocytochemistry and reconstruction of neuron morphology is performed. ***B***, Representative images of EB3-GFP puncta tracks from Pten WT neurons (top panels), Pten-KO neurons (middle panels), and Pten-Raptor DKO neurons (bottom panels). White outlines show edge of cell. ***C***, PtRap-DKO does not inhibit Pten-KO-mediated increases in MT polymerization velocity (Brown–Forsythe and Welch ANOVA tests: WT vs Pten-KO *p* = 0.0004, WT vs PtRap-DKO *p* < 0.0001, Pten-KO vs PtRap-DKO *p* = 0.5172). ***D***, Representative images of Pten WT neurons (top panel), Pten-KO neurons (middle panel), and Pten-Raptor DKO neurons (bottom panel) immunolabeled with beta-III-tubulin and 2D reconstructions for Sholl analysis. ***E***, PtRap-DKO does not inhibit Pten-KO-mediated increases in Sholl of intersections (2-way ANOVA, Tukey’s multiple-comparisons tests: WT vs Pten-KO *p* < 0.0001, WT vs PtRap-DKO *p* = 0.1894, Pten-KO vs PtRap-DKO *p* = 0.2186). ***F***, PtRap-DKO does not inhibit Pten-KO-mediated increases in Sholl of length (2-way ANOVA, Tukey’s multiple-comparisons tests: WT vs Pten-KO *p* = 0.0024, WT vs PtRap-DKO *p* = 0.1171, Pten-KO vs PtRap-DKO *p* = 0.9281). ***G***, PtRap-DKO does not inhibit Pten-KO-mediated increases in soma size (Brown–Forsythe and Welch ANOVA tests: WT vs Pten-KO *p* = 0.0031, WT vs PtRap-DKO *p* < 0.0001, Pten-KO vs PtRap-DKO *p* = 0.0405) ***H***, PtRap-DKO does not inhibit Pten-KO-mediated increases in total neurite length (Brown–Forsythe and Welch ANOVA tests: WT vs Pten-KO *p* = 0.0032, WT vs PtRap-DKO *p* = 0.4118, Pten-KO vs PtRap-DKO *p* = 0.9981).

**Figure 3.**
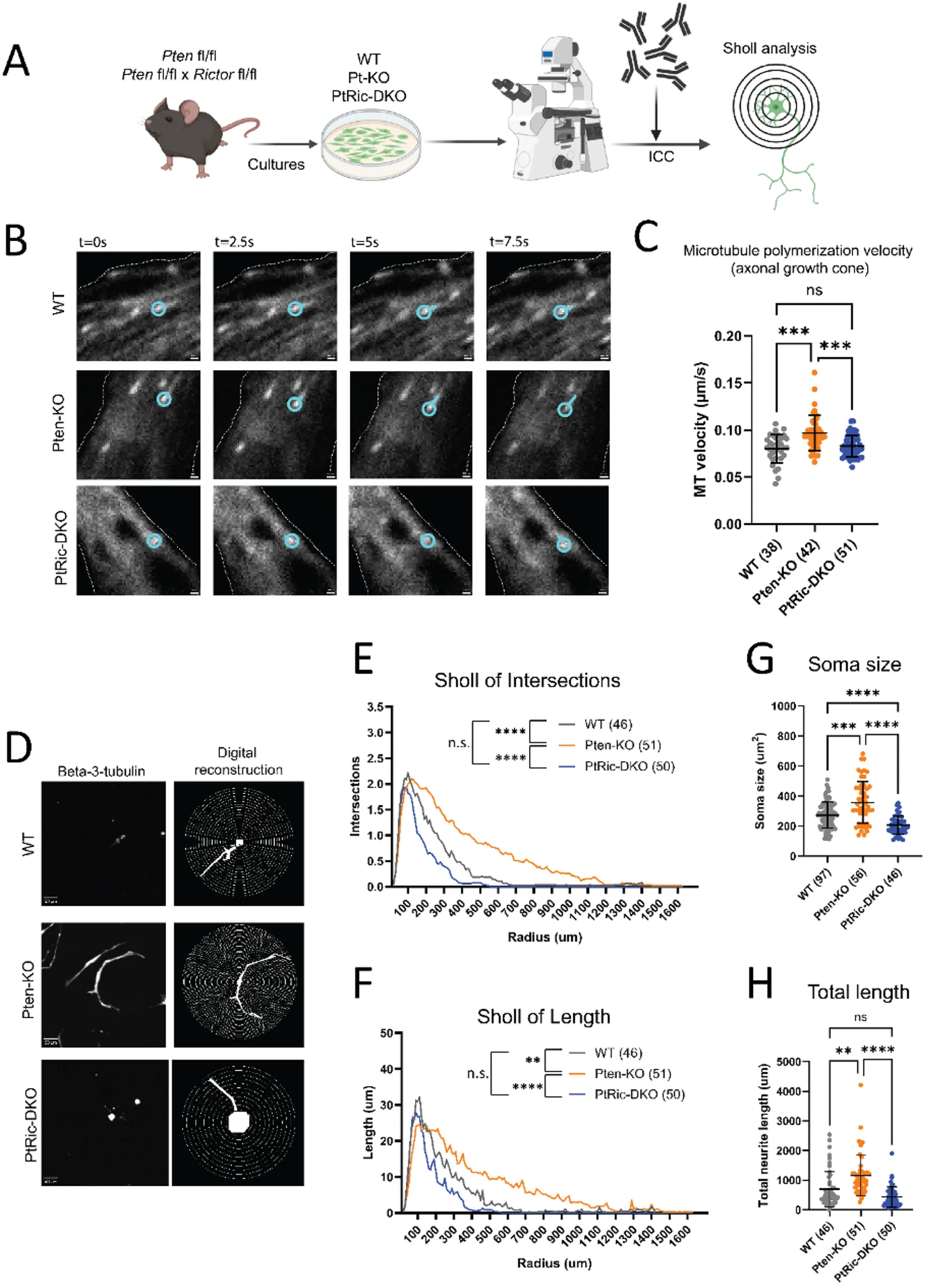
Genetic disruption of mTORC2 inhibits Pten-KO-mediated axon growth in vitro. Codeletion of *Pten* and *Rictor* in cultured DRG neurons results in reversal of *Pten-*KO-mediated increase in MT polymerization velocities and immunostaining for Beta-III-tubulin reveals decreased axon outgrowth by quantitative Sholl analysis compared with Pten-KO neurons. ***A***, Experimental design. Dorsal root ganglia are harvested fresh from adult *Pten*^fl/fl^ animals or adult *Pten*^fl/fl^ *Rictor*^fl/fl^ animals and primary sensory neurons are then dissociated and grown in primary culture. At day in vitro (DIV) 4, the neurons are transfected with a plasmid encoding EB3-GFP to label plus-end microtubules and Cre recombinase (pRubi-C-T2A-Cre) to conditionally delete *Pten* and *Rictor* or EB3-GFP and RFP (RedRubi) as negative controls (*Pten* WT). At DIV 6–7 the cultures are live-imaged to generate movies of EB3-GFP puncta movement. After imaging, the cells are fixed and subsequent immunocytochemistry and reconstruction of neuron morphology is performed. ***B***, Representative images of EB3-GFP puncta tracks from *Pten* WT neurons (top panels), *Pten-*KO neurons (middle panels), and *Pten-Rictor* DKO neurons (bottom panels). White outlines show edge of cell. ***C***, PtRic-DKO inhibits Pten-KO-mediated increases in MT polymerization velocity (Brown–Forsythe and Welch ANOVA tests: WT vs Pten-KO *p* = 0.0004, WT vs PtRic-DKO *p* = 0.9442, Pten-KO vs PtRic-DKO *p* = 0.0005). ***D***, Representative images of Pten WT neurons (top panel), Pten-KO neurons (middle panel), and Pten-Rictor DKO neurons (bottom panel) immunolabeled with beta-III-tubulin and 2D reconstructions for Sholl analysis. ***E***, PtRic-DKO does not inhibit Pten-KO-mediated increases in Sholl of intersections (2-way ANOVA, Tukey’s multiple-comparisons tests: WT vs Pten-KO *p* < 0.0001, WT vs PtRic-DKO *p* = 0.1011, Pten-KO vs PtRic-DKO *p* < 0.0001). ***F***, PtRic-DKO does not inhibit Pten-KO-mediated increases in Sholl of length (2-way ANOVA, Tukey’s multiple-comparisons tests: WT vs Pten-KO *p* = 0.0024, WT vs PtRic-DKO *p* = 0.1218, Pten-KO vs PtRic-DKO *p* < 0.0001). ***G***, PtRic-DKO inhibits Pten-KO-mediated increases in soma size (Brown–Forsythe and Welch ANOVA test: WT vs Pten-KO *p* = 0.0031, WT vs PtRic-DKO *p* < 0.0001, Pten-KO vs PtRic-DKO *p* = 0.0006). ***H***, PtRic-DKO inhibits Pten-KO-mediated increases in total neurite length (Brown–Forsythe and Welch ANOVA tests: WT vs Pten-KO *p* = 0.0032, WT vs PtRic-DKO *p* = 0.0506, Pten-KO vs PtRic-DKO *p* < 0.0001).

**Figure 4.**
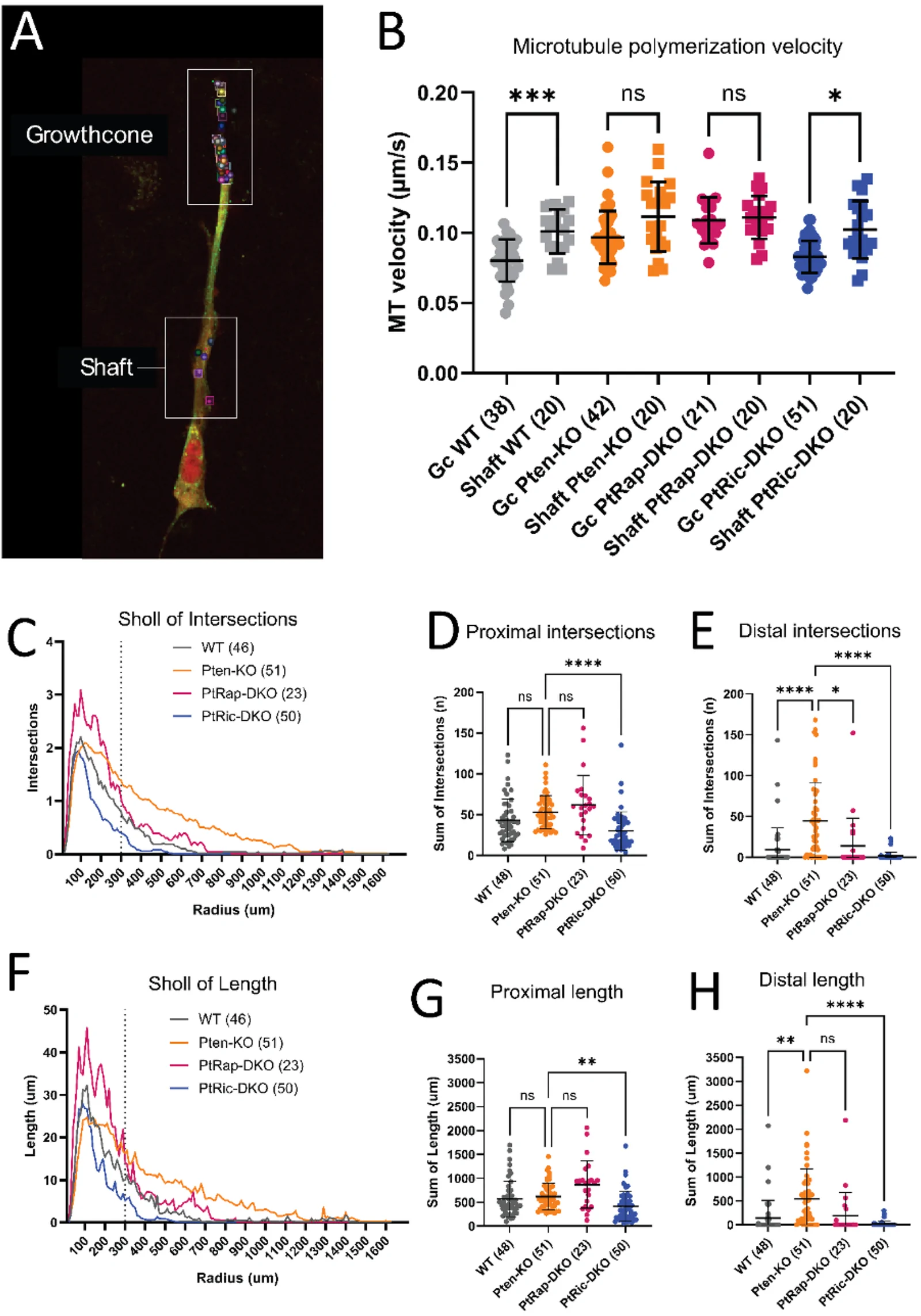
Subcellular regional differences in microtubule polymerization. Analysis of adult dorsal root ganglion neuron cytoskeletal dynamics and growth patterns in cultured neurons in the growth cone versus the axon shaft demonstrates differential regulation by *Pten*, mTORC1, and mTORC2*.* ***A***, Representative image of cultured DRG neuron after automated EB3-GFP puncta-tracking with growth cone and axon shaft regions of interest outlined. ***B***, MT polymerization velocities are significantly increased in the shaft compared with the growth cone in *Pten* WT neurons. After *Pten*-KO, MT polymerization velocity increases in the growth cone compared with WT neurons, but MT polymerization velocity is not significantly increased in the shaft compared with WT. PtRap*-*DKO does not reverse *Pten*-KO-mediated changes in MT polymerization velocity in the growth cone or shaft. PtRic-DKO reverses Pten-KO-mediated MT polymerization velocity increase in growth cone by not the shaft. Overall, changes in MT polymerization velocities in the axon shaft were significantly increased compared with the axon growth cone and were not impacted by genetic disruption of *Pten* signaling. For quantitative results and statistical comparisons, see Extended Data Figures 4-1, 4-2. ***C***, Sholl of intersection analysis comparing WT, *Pten*-KO, PtRap-DKO, and PtRic-DKO demonstrates a change in the rank-order of number of intersections by genetic condition at 300 μm, where PtRap-DKO falls below *Pten*-KO. ***D***, Sum of intersection analysis for DRG neurons at a radius below 300 μm (proximal intersections) demonstrates a significant decrease in sum of intersections for PtRic-DKO compared with all other conditions. ***E***, Sum of intersection analysis for DRG neurons at a radius above 300 μm (distal intersections) demonstrates a significant decrease in sum of intersections for both PtRap-DKO and PtRic-DKO compared with Pten-KO. For quantitative results and statistical comparisons, see Extended Data Figure 4-3. For quantitative results and statistical comparisons for ***D***, ***E***, see Extended Data Figures 4-3, 4-4. ***F***, Sholl of length analysis comparing WT, Pten-KO, PtRap-DKO, and PtRic-DKO demonstrates a change in the rank-order of number of intersections by genetic condition at 300 μm, where PtRap-DKO falls below Pten-KO. ***G***, Sum of length analysis for DRG neurons at a radius below 300 μm (proximal length) demonstrates a significant decrease in sum of lengths for PtRic-DKO compared with all other conditions. For quantitative results and statistical comparisons, see Extended Data Figure 4-5. ***H***, Sum of length analysis for DRG neurons at a radius above 300 μm (distal length) demonstrates a significant increase in sum of lengths for Pten-KO compared with WT and PtRic-DKO but not PtRap-DKO. For quantitative results and statistical comparisons for ***G***, ***H***, see Extended Data Figures 4-5, 4-6.

10.1523/ENEURO.0116-26.2026.f4-1 Figure 4-1 Data represented in Fig. 4B. Average polymerization velocity within subaxonal regions. “Distal” refers to the axonal growth cone, and “proximal” refers to the proximal region of the axon shaft. Download Figure 4-1, DOCX file.

10.1523/ENEURO.0116-26.2026.f4-2 Figure 4-2 Statistical analyses of data represented in Fig. 4B. Brown-Forsythe and Welch ANOVA test with Dunnett’s T3 multiple comparisons of average polymerization velocity within subaxonal regions. N cells shown in parentheses after each condition. “Gc” refers to distal growth cone, “Shaft” refers to proximal axon shaft. Significant comparisons bolded. Download Figure 4-2, DOCX file.

10.1523/ENEURO.0116-26.2026.f4-3 Figure 4-3 Data represented in Fig. 4D-E. Sum of proximal (radius≤350um) and distal (radius>350um) Sholl of intersections across genetic conditions. Sholl of intersections within designated regions was summed per cell, then averaged across cells per genetic condition. Download Figure 4-3, DOCX file.

10.1523/ENEURO.0116-26.2026.f4-4 Figure 4-4 Brown-Forsythe and Welch ANOVA tests of sums of proximal (radius≤300um) and distal (radius>300um) Sholl of intersections across genetic conditions. Sholl of intersections within designated regions was summed per cell, then averaged across cells per genetic condition. Significant comparisons are bolded. Download Figure 4-4, DOCX file.

10.1523/ENEURO.0116-26.2026.f4-5 Figure 4-5 Data represented in Fig. 4G-H. Sum of proximal (radius≤300um) and distal (radius>300um) Sholl of length across genetic conditions. Sholl of length within designated regions was summed per cell, then averaged across cells per genetic condition. Download Figure 4-5, DOCX file.

10.1523/ENEURO.0116-26.2026.f4-6 Figure 4-6 Brown-Forsythe and Welch ANOVA tests of sums of proximal (radius≤300um) and distal (radius>300um) Sholl of length across genetic conditions. Sholl of length within designated regions was summed per cell, then averaged across cells per genetic condition. Significant comparisons are bolded. Download Figure 4-6, DOCX file.

#### Automated puncta-tracking

Live-imaged cells were processed using an automated puncta-tracking pipeline we developed in the software arivis4D, from Zeiss. The full pipeline and documentation is publicly available at https://github.com/smevans19/puncta-tracking-arivis. In brief, the pipeline consists of two broad steps: segmenting (puncta identification) and tracking (identifying puncta movement over time).

Segmenting was performed by performing bilateral denoising, then using arivis’ automated “blob finder” algorithm to identify fluorescent puncta (i.e., labeled MT plus-ends) according to a user-determined probability threshold. Finally, we applied a size-based exclusion filter to reduce segments below a user-determined area threshold, reducing false positives and background noise detection.

Tracking was performed by importing the segments generated during the previous step, then using arivis’ “Tracking” algorithm to link segment centroids across consecutive frames, with linear regression-based trajectories. One important user-determined parameter was the max distance traveled across frames, which was calculated by measuring the distance traveled by a visibly fast-moving punctum across two timepoints, for 10 sets of timepoints distributed across the imageset, then averaging these distances, and rounding to the nearest 25 nm. Weighting was biased toward motion, rather than geometric feature, since MTs follow consistently linear paths (motion), but the appearance of a labeled MT (feature) can vary widely across frames due to imaging variability.

Finally, track data was exported and used to calculate MT polymerization velocity for each track. Track velocity was then averaged, and average velocity per cell is reported. More details and documentation for the arivis segmenting/tracking pipeline can be found at https://github.com/smevans19/puncta-tracking-arivis.

## Results

### Novel automated microtubule tracking pipeline is accurate and efficient

In this study, we use a primary culture system of peripheral sensory neurons to study microtubule (MT) dynamics in vitro. Specifically, we culture neurons from the dorsal root ganglia of adult mice, use transfection with EB3-GFP to fluorescently label MT plus-ends, and then live-image cells over a short period of time (120 frames at ∼0.79 fps) to visualize MT polymerization dynamics (Fig. 1*A*). EB3 (end-binding protein 3) binds to the actively polymerizing plus-ends, so EB3-GFP labeling provides a visual readout of MT polymerization. To quantify MT velocity, the fluorescent puncta (GFP-labeled MT plus-ends) are tracked across consecutive frames of the imageset. However, manually tracking a single cell, which can contain hundreds of MT tracks (Fig. 1*C*), takes multiple hours per cell, limiting data throughput. We therefore constructed an automated image-processing pipeline using arivis4D (Zeiss) to track MT movement in these image sequences. Automated tracking using the pipeline (Fig. 1*D*) takes roughly 20–30 min per cell, massively improving processing efficiency.

To prepare images for automated tracking, we first identified a region of interest (the axonal growth cone; Fig. 1*B_1_*) and then denoised images with a bilateral filter at a user-defined radius (Fig. 1*B_2_*). This decreases background noise to target GFP puncta (MT plus-ends) with minimal loss of information. Then, our pipeline uses a series of Gaussian smoothing kernels (based on a user-defined diameter of interest) and a user-determined probability threshold to identify regions of increased fluorescent intensity, which segments (i.e., digitizes) the GFP puncta out from the rest of the image (Fig. 1*B_3_*). A watershed algorithm is used to individuate adjacent/touching segments. These digital segments are tracked across frames (Fig. 1*B_4_*), using a linear regression model based on the center of geometry for each segment (each MT plus-end). This quantification method produces a rich dataset describing MT plus-end movement over time. The velocity of all MT tracks per image set is then averaged and reported as microtubule polymerization velocity per cell.

Before using the pipeline to quantify experimental data, we first validated pipeline accuracy. We processed the same 10 wild-type neurons both manually (using the mTrackJ plugin in ImageJ) and with the automated pipeline. There was no significant difference in the resulting velocities from manual (0.07374 ± 0.008977 μm/s) compared with automated (0.08415 ± 0.01341 μm/s) processing (Fig. 1*E*). We also find that, overall, manual puncta-tracking produces fewer (*n* tracks = 114.9 ± 70.77), but longer (track length = 0.6128 ± 0.1576 μm), MT tracks, while automated tracking produces more (*n* tracks = 336.3 ± 265.3), shorter (track length = 0.2435 ± 0.07774 μm) tracks (Extended Data Fig. 1-1). This did not result in significant velocity differences when tracks were averaged over a growth cone, as in our dataset, but it may be an important consideration for other applications.

After validating pipeline accuracy to manual tracking, we then sought to validate the pipeline’s ability to detect potential differences in MT velocity across experimental conditions, using both pharmacological and genetic manipulations. First, we intentionally reduced MT velocity by acute in vitro treatment with vinblastine, a chemotherapy drug which is known to decrease MT polymerization velocity by binding directly to the beta-tubulin subunit of tubulin dimers, causing them to aggregate into nonfunctional spiral polymers instead of forming normal MTs (Toso et al., 1993; Dhamodharan et al., 1995; Gigant et al., 2005). A previous study in neonatal hippocampal neuronal cultures found acute vinblastine treatment significantly reduced MT polymerization velocity in vitro (Getz et al., 2022)*.* We treated DRG cell cultures with 2 nM vinblastine and imaged them 30–120 min posttreatment. Ten of these image sets were then quantified using both manual and automated puncta-tracking. Manual tracking found 2 nM vinblastine treatment reduced polymerization velocity to 0.05709 ± 0.01878 μm/s; automated tracking of the same cells found 2 nM vinblastine treatment reduced polymerization velocity to 0.06833 ± 0.01336 μm/s, with no statistically significant difference between the two processing methods (Fig. 1*E*). This confirmed that the automated pipeline was able to detect a known inhibition in polymerization velocity. Next, we used a genetic manipulation to intentionally increase MT polymerization rates. Previous work (Getz et al., 2022) has found that Pten-KO significantly increased MT polymerization rates in the axonal growth cone of embryonic hippocampal neurons. After culturing, we generated *Pten*-KO neurons and found no significant difference between velocities generated by manual and automated puncta-tracking (0.1041 ± 0.03067 μm/s vs 0.1036 ± 0.01488 μm/s, *p* = 0.9631; Fig. 1*E*).

We were therefore satisfied that our automated tracking pipeline is both accurate relative to the current standard of quantification (manual puncta-tracking) and sensitive enough to detect subtle differences between MT polymerization rates at varying experimental conditions.

### *Pten*-KO in vitro increases MT polymerization rates, soma size, axon length, and neurite branching in peripheral sensory neurons

*Pten* is known to regulate the MT cytoskeleton and cellular growth, though the precise effects on MT dynamics and axonal growth are not clear. We generated primary cultures from *Pten^fl/fl^* mice (Fig. 2*A*) and used our automated tracking pipeline to quantify MT polymerization velocity (Fig. 2*B*), as previously described in a further three sets of cultures. We found that *Pten*-KO significantly increases MT polymerization rates in the axonal growth cone (WT = 0.08024 ± 0.01494 μm/s, Pten-KO = 0.09680 ± 0.01883 μm/s, *p* = 0.0004; Fig. 2*C*). After live imaging, cultures were fixed and stained for beta-3-tubulin to visualize neurite outgrowth and soma size (Fig. 2*D*). Fixed cell images were digitally reconstructed, and Sholl analysis was performed to quantify changes in neurite morphology, including length and branching complexity (Fig. 2*D*). We found that *Pten*-KO significantly increases Sholl of intersections and Sholl of length over WT neurons (Fig. 2*E*,*F*), as well as total neurite length (WT = 694.7 ± 596.3 μm, Pten-KO = 1157 ± 691.9 μm, *p* = 0.0032; Fig. 2*H*). Pten-KO increases the average intersections and length specifically at radii further from the soma, which describes a pattern of neurite elongation, rather than increased branching complexity. These morphological changes are described in more detail later in this study (Fig. 4). Finally, in addition to changes in neurite morphology, *Pten*-KO significantly increases soma size over WT neurons (WT = 274.1 ± 86.26 μm^2^, Pten-KO = 358.1 ± 138.7 μm^2^, *p* = 0.0031; Fig. 2*G*). These increases in Sholl of intersections, Sholl of length, and soma size reflect a morphological phenotype of substantially increased soma size and neurite length compared with WT control neurons and are correlated with the increased MT velocity we observed in the same cells.

### mTORC1 loss of function does not reverse hypertrophic effects of *Pten-*KO in peripheral sensory neurons

We next wanted to investigate the downstream signaling pathway by which *Pten*-KO drives MT polymerization. Specifically, we focused on two primary downstream signaling targets: mTORC1, which is dependent on the protein RAPTOR, and mTORC2, dependent on RICTOR. Knocking out *Pten* causes upregulation of both mTORC1 and mTORC2 (Y. Liu et al., 2015; Tariq et al., 2022), so we hypothesized that a codeletion of either of these complexes (alongside PTEN loss) may potentially reverse the increased MT polymerization rates.

To investigate the effects of the mTORC1 signaling pathway, we generated primary neuronal cultures from *Pten^fl/fl^* × *Raptor^fl/fl^* mice, and transfected with either a Cre-containing plasmid to yield *Pten*-KO × *Raptor*-KO cultures (PtRap-DKO), or a control plasmid to yield WT neurons. DRG neurons were cultured from adult mice (Fig. 2*A*) and live-imaged as previously described on DIV6–7 to visualize MT dynamics (Fig. 2*B*). We found that knocking out *Raptor* (mTORC1) did not reverse increased MT polymerization rates caused by *Pten*-KO (PtRap-DKO = 0.1089 ± 0.01638 μm/s, WT vs PtRap-DKO *p* < 0.0001, Pten-KO vs PtRap-DKO *p* = 0.5172). PtRap-DKO cells had significantly increased polymerization velocity from WT levels, similar to that of *Pten*-KO cells (Fig. 2*D*). We next quantified the effect of this codeletion on neuronal morphology and found that knocking out *Raptor* did not decrease Sholl of intersections (Pten-KO vs PtRap-DKO *p* = 0.2186), Sholl of length (Pten-KO vs PtRap-DKO *p* = 0.9281), soma size (PtRap-DKO = 431.8 ± 149.2 μm^2^, WT vs PtRap-DKO *p* < 0.0001, Pten-KO vs PtRap-DKO *p* = 0.0405), or total neurite length (PtRap-DKO = 1,061 ± 914.4 μm, WT vs PtRap-DKO *p* = 0.4118, Pten-KO vs PtRap-DKO *p* = 0.9981) from elevated *Pten*-KO levels (Fig. 2*E*–*H*). We did observe different patterns of increased neurite extension and branching between *Pten*-KO and PtRap-DKO cells at varying distances from the cell body (Fig. 2*E*,*F*), which we investigate later in this study (Fig. 4).

### mTORC2 loss of function reverses *Pten-*KO-mediated hypertrophy in peripheral sensory neurons

We next investigated the effects of the mTORC2 signaling pathway. We cultured DRG neurons from *Pten^fl/fl^* × *Rictor^fl/fl^* mice, transfected with a Cre-containing plasmid to generate *Pten*-KO × *Rictor*-KO (PtRic-DKO) cells or a control plasmid to generate WT neurons, and live-imaged to quantify MT polymerization rates as previously described (Fig. 3*A*,*B*). Codeletion of Rictor appeared to fully reverse the accelerated MT polymerization rates in *Pten-*KO neurons: PtRic-DKO cells demonstrated MT velocity rates similar to WT levels, significantly decreased from *Pten*-KO levels (PtRic-DKO = 0.08287 ± 0.01133 μm/s, WT vs PtRic-DKO *p* = 0.9442, Pten-KO vs PtRic-DKO *p* = 0.0005; Fig. 3*C*). Similarly, *Rictor*-KO appeared to reverse the morphological changes observed in *Pten*-KO neurons. We fixed and stained cells to quantify cellular outgrowth (Fig. 3*D*) as previously described. We found PtRic-DKO cells had similar Sholl of intersections and length compared with WT cells, decreased from that of Pten-KO cells (Fig. 3*E*,*F*). We also found that *Rictor*-KO reduces soma size (PtRic-DKO = 207.0 ± 61.06 μm^2^, WT vs PtRic-DKO *p* < 0.0001, Pten-KO vs PtRic-DKO *p* = 0.0006; Fig. 3*G*) and total neurite length (PtRic-DKO = 430.8 ± 342.2 μm, WT vs PtRic-DKO *p* = 0.0506, Pten-KO vs PtRic-DKO *p* < 0.0001; Fig. 3*H*) from elevated *Pten*-KO levels to near-WT levels. Therefore, we conclude that mTORC2 mediates *Pten*-KO’s effects on cytoskeletal dynamics and neuronal morphology in peripheral sensory neurons.

### Pten differentially regulates neuronal growth across subcellular regions

We observed that, even though *Pten*-KO and PtRap-DKO cells have similar MT polymerization velocities, soma sizes, and total neurite length, there appeared to be differences between the two conditions with respect to patterns of neurite morphology. PtRap-DKO cells had increased Sholl of intersections and Sholl of length compared with WT controls primarily in the proximal portions of neurites (radius <300 μm), while Pten-KO cells demonstrated increases in distal portions (radius >300 μm; Fig. 2*E*,*F*). We hypothesized that this difference may reflect subcellular localization of MT regulation pathways. Previously, we quantified MT polymerization velocity in the axonal growth cone, the most distal point of the axon. We next quantified MT polymerization velocity in the axonal shaft and investigated whether this velocity was correlated with proximal overgrowth in PtRap-DKO neurons.

Automated puncta-tracking was performed on the same set of images, but instead of quantifying MT dynamics in the axonal growth cone, the ROI was shifted to the proximal axon shaft (Fig. 4*A*). We found that both the absolute velocities and the relative changes that occur are different in the proximal axon shaft compared with the axonal growth cone across all four genetic conditions. Specifically, MT polymerization velocity in WT cells is nonsignificantly increased in the proximal axon shaft compared with the growth cone, with no significant difference between WT and *Pten*-KO velocities in the proximal axon shaft (Fig. 4*B*; Extended Data Figs. 4-1, 4-2). Interestingly, there was no significant difference between *Pten*-KO and *PtRic*-DKO MT polymerization velocities within the axon shaft (Fig. 4*B*; Extended Data Figs. 4-1, 4-2). This is in apparent contrast to our findings in the growth cone, where PtRic-DKO significantly reduced polymerization rates from *Pten*-KO, implying region-specific differences in polymerization or regulation.

Next, we further quantified patterns of neuronal outgrowth in these cells by summing proximal (radius <300 μm) and distal (radius >300 μm) intersections and length, as there appeared to be a rank-order change between *Pten-*KO and PtRap-DKO at approximately a 300 μm radius in our Sholl analyses (Fig. 4*C*,*F*). Neither *Pten*-KO nor PtRap-DKO neurons had significantly different proximal intersections or length compared with WT neurons (Fig. 4*D*,*G*; numerical results in Extended Data Figs. 4-3–4-6). However, at more distal locations, *Pten*-KO had significantly increased sum of intersections and length compared with WT neurons and significantly increased sum of length compared with PtRap-DKO neurons (Fig. 4*E*,*H*; numerical results in Extended Data Figs. 4-3–4-6). In other words, *Pten*-KO significantly increased growth at the most distal points of the neurite but does not increase branching or arborization at locations more proximal to the cell body. In contrast, PtRap-DKO neurons did not have significantly longer individual neurites than WT neurons. Instead, the observed increase in total neurite length (Fig. 2*H*) seems to be due to a nonsignificant increase in proximal branching and neurite complexity (Fig. 4*D*,*G*). Finally, PtRic-DKO neurons demonstrated significant decreases compared with *Pten-*KO neurons in sum of intersections and length at all distances, proximal and distal. This contrasts with the observed elevated MT polymerization rates in PtRic-DKO axon shafts and suggests that MT-driven mechanisms of axon growth may be region specific. Overall, these results highlight potential differences in *Pten*-driven regulation of growth and MT dynamics across subaxonal regions.

## Discussion

Peripheral neuropathy is a serious, rapidly growing public health issue, with severe impacts to quality of life and poor clinical outcomes (Feldman et al., 2019; Burgess et al., 2021). Earlier in vivo studies on peripheral nerve regeneration have found knocking out the tumor suppressor gene *Pten* strongly promotes axonal outgrowth and functional recovery after injury (Park et al., 2008; Christie et al., 2010; Kurimoto et al., 2010; Liu et al., 2010; Ohtake et al., 2014; Singh et al., 2014; Walker and Xu, 2014); however, *Pten* deletion is associated with human tumor syndromes and cancers (Hollander et al., 2011; Yehia et al., 2023), making it a challenging clinical target. Here, we performed double-knockouts in primary cultures of DRG neurons with *Pten* and either of its immediate downstream effectors, *Raptor* or *Rictor*, to better understand which *Pten*-regulated cellular processes directly support axonal growth.

We found that in cultures of peripheral sensory neurons, *Pten*-KO both accelerated MT polymerization rates in the axonal growth cone and increased distal neurite hypertrophic morphology (Figs. 2*C*, 4*E*,*H*). Although prior studies have established that *Pten* deletion promotes outgrowth in several neuronal subtypes, including retinal (Park et al., 2008), corticospinal (Liu et al., 2010), and peripheral neurons (Christie et al., 2010), these studies concluded that this regeneration was due to *Pten*’s regulation of protein translation and made little or no mention of possible cytoskeletal effects. Our observations of MT polymerization after *Pten* loss of function and our quantification of the resulting impacts on neuronal morphology reveal a novel downstream molecular consequence of *Pten*-KO, which may promote axon extension and regeneration independently from *Pten*’s regulation of protein synthesis. Importantly, our work focused on adult dorsal root ganglion neurons, reflecting a preclinical model that is relevant to peripheral neuropathy in patients. Overall, our results suggest manipulating MT cytoskeletal dynamics may be an alternate therapeutic strategy to Pten-KO for promoting nerve regeneration in a clinical setting.

To better understand the molecular relationship between Pten signaling and cytoskeleton dynamics, we focused on two immediate downstream targets of Pten: mTORC1, which is Raptor-dependent, and mTORC2, which is Rictor-dependent. mTORC1 and mTORC2 are both positive regulators of cell growth which are upregulated when Pten is knocked out (P. Liu et al., 2015; Chen et al., 2019; Tariq et al., 2022). In this study, we leveraged a conditional knock-out approach, using transfection of a Cre recombinase to codelete either mTORC1 or mTORC2 in addition to Pten. Using an in vitro codeletion system allowed us to compare *Pten*-KO × *Rictor*-KO and *Pten*-KO × *Raptor*-KO neurons with control (WT) neurons from the same culture conditions.

First, we found knocking out *Raptor* (mTORC1) reduced distal neurite outgrowth but did not fully reverse the effects of *Pten*-KO on the MT cytoskeleton with regard to polymerization velocity (Fig. 2*C*). Knocking out Raptor also did not significantly impact somal hypertrophy compared with *Pten*-KO neurons (Fig. 2*G*). This was a surprising finding, as previous studies have shown that both conditional codeletion of mTORC1 in vivo and direct pharmacological inhibition of mTORC1 by rapamycin were sufficient to rescue *Pten* loss-mediated phenotypes of neuronal hypertrophy and aberrant migration in neonatal mice (Getz et al., 2016; Tariq et al., 2022). Likewise, in human neural organoids, either mTORC1-KO or mTORC2-KO independently was sufficient to rescue hypertrophy and electrical hyperactivity caused by Pten-KO (Dhaliwal et al., 2024). While these earlier findings support a role for mTORC1 in regulation of neuronal hypertrophy, they may be context specific: neonatal mice and brain organoids model early CNS development, not adult peripheral nerve regeneration, as in our study. Additionally, mTORC1 is thought to regulate cell growth via regulating protein translation (Laplante and Sabatini, 2009; Takei and Nawa, 2014; Saxton and Sabatini, 2017) and may therefore not have an immediate observable impact on cytoskeleton dynamics. Finally, our experimental system has genetic knockouts performed in vitro, and cells are live-imaged 2–4 d after the knockouts are performed, in contrast to the much longer timecourse of previous in vivo models (Getz et al., 2016; Tariq et al., 2022). An important future experiment would be to study the effects of *PtRap-*DKO in an in vivo model of peripheral nerve injury and regeneration, since our in vitro culture system may have obscured the long-term effects of Raptor on neuronal regeneration.

In the present study, codeletion of Rictor (mTORC2) reversed the increased neurite length, neurite branching, and soma size of PTEN-KO (Fig. 3). This aligns with a previous study showing mTORC2-KO rescued morphological effects of Pten-KO in dentate gyrus neurons, particularly effects distal to the cell body (e.g., arborization and branching), but not more proximal effects (soma size or number of primary dendrites; Cullen et al., 2023). We also observed that mTORC2 altered growth cone MT dynamics from Pten-KO, significantly reducing MT polymerization rates. Existing literature has established a strong link between mTORC2 and the actin cytoskeleton (Laplante and Sabatini, 2009; Angliker and Rüegg, 2013; Huang et al., 2013), but the effects of mTORC2 on the microtubule cytoskeleton have not been well studied. mTORC2 may affect the MT cytoskeleton through either of two potential downstream factors: CRMP2, which binds to tubulin heterodimers, promotes polymerization and plays a role in axonal growth cone guidance (Inagaki et al., 2001; Fukata et al., 2002), and MAP1B, which binds to dynamic (tyrosinated) microtubules and thus regulates MT polymerization dynamics (Bouquet et al., 2004; Kawauchi and Hoshino, 2007; Tymanskyj et al., 2012). Activity in this pathway due to *Pten*-KO would, in theory, cause an increase in stable, detyrosinated MTs and an increase in CRMP2 expression (which would then promote polymerization). Indeed, *Pten*-KO can increase levels of stable (detyrosinated) microtubules, and *Pten*-KO driven hypertrophy can be reversed through destabilizing (tyrosinating) the MTs directly (Kath et al., 2018). Both the CRMP2- and MAP1B-driven polymerization effects would theoretically be reversed by *Rictor* (mTORC2) knock-out, which could explain our observed decrease in MT polymerization rates. However, as our study did not directly observe signaling molecules in between mTORC2 and MT polymerization, the exact intermediary mechanism remains unresolved and warrants future study.

A secondary finding is that, while knocking out mTORC1 (*Raptor*) reversed Pten-KO’s effects on distal axonal outgrowth, these *Pten*-KO × *Raptor*-KO codeleted cells had increased proximal neurite branching when compared with both WT and *Pten*-KO cells. This observed difference in subcellular regional regulation of axon growth prompted us to quantify MT polymerization in the proximal axon shaft and assess whether the actions of *Pten* and its downstream effector were distinct here compared with the growth cone. We found that MT polymerization rates in the proximal shaft did not significantly vary across any genetic conditions and was relatively high compared with the axonal growth cone. This raises questions on subcellular regional differences in MT regulation. Most past studies on MT polymerization dynamics in neurons have been focused on the axonal growth cone: peripheral neuropathy usually starts distally and progresses inward, and the growth cone is the driving region in regeneration and development, with many vital growth-modulating proteins concentrating there. For example, MAP1B localizes to the axonal growth cone (Black et al., 1994; Bush et al., 1996; Mack et al., 2000; Inagaki et al., 2001). Our findings agree that *Pten* selectively regulates MTs in the axonal growth cone, but not the axon shaft. One possible mechanism may be that *Pten* exerts a primary effect on axonal actin polymerization which then indirectly affects MT polymerization rates. Whether the regional differential regulation is due to compartmentalization of PTEN, tubulin abundance, the effects of axon transport, or a different unknown mechanism is unclear and is a question of interest for future experiments. To our knowledge, there are no previous studies that have examined Pten-mediated MT polymerization changes across axon compartments in the CNS. The MT cytoskeleton appears to have differential patterns of polymerization in axons versus dendrites in cultured hippocampal neurons (Kollins et al., 2009), but whether MT polymerization varies within the different regions of the axon or whether these distinct processes are regulated by Pten is unknown.

The MT cytoskeleton is vital to cellular growth and function, as it plays a variety of structural and transport roles, and is a driving force behind axon outgrowth. Here, we find that *Pten* regulates MT cytoskeletal dynamics in the axonal growth cone, but not the shaft, and that this regulation occurs through mTORC2 and not mTORC1. Identifying a region-specific correlation between MT polymerization rates and axonal outgrowth is a novel finding and supports a growth cone-specific pathway of MT polymerization regulation via mTORC2. Our work to elucidate this pathway supports existing literature that MT polymerization in the axonal growth cone may drive outgrowth in peripheral sensory neurons and highlights the MT cytoskeleton as a regenerative therapeutic target for peripheral neuropathy.

## Synthesis

Reviewing Editor: Jennifer Dulin, Texas A&M University Decisions are customarily a result of the Reviewing Editor and the peer reviewers coming together and discussing their recommendations until a consensus is reached. When revisions are invited, a fact-based synthesis statement explaining their decision and outlining what is needed to prepare a revision will be listed below. The following reviewer(s) agreed to reveal their identity: NONE.

Both reviewers agree this is a well-conceived, technically solid study that uses an appropriate conditional co-deletion strategy and a well-validated EB3 tracking pipeline to establish mTORC2, rather than mTORC1, as the key mediator of PTEN-regulated microtubule polymerization and growth in adult DRG neurons. However, both raise substantive concerns that the claims are overstated relative to the data-particularly the use of "rescue," the translational/therapeutic framing in the absence of in vivo or injury-model evidence, and the still-speculative downstream link between mTORC2 and microtubule regulation (e.g., AKT/GSK3β/CRMP2). Revisions should focus on tempering interpretation, clearly defining and disambiguating outcome measures (hypertrophy vs. neurite outgrowth vs. axonal elongation vs. complexity), strengthening causal evidence for the microtubule-dependence of PTEN-driven growth, and improving reporting of statistics, methodological details, and pipeline reproducibility.

Reviewers' comments:

Reviewer #1:

The manuscript is well written, logically organized, and technically solid. The authors clearly define the question and use appropriate experiments to show that PTEN regulates growth cone microtubule polymerization primarily through mTORC2 rather than mTORC1 in adult DRG neurons. The conditional co-deletion strategy is appropriate, and the automated EB3 tracking pipeline is carefully validated, which strengthens confidence in the results.

My main concern is that the significance and translational implications are overstated. The study is entirely based on in vitro DRG cultures and does not include an injury model, in vivo regeneration, or functional recovery. Therefore, statements describing microtubules as a strong therapeutic target for peripheral regeneration should be toned down.

A second concern is that the mechanistic pathway remains incomplete. While the data support that mTORC2, but not mTORC1, is required for PTEN-mediated regulation of microtubule dynamics, the downstream link between mTORC2 and microtubule regulation is not directly tested. Molecules such as AKT, GSK3β, and CRMP2 are only discussed speculatively, so the conclusions should be more cautious.

In addition, the claim that PTEN-driven neurite growth depends on microtubule activation is still largely correlative. Vinblastine is used only for pipeline validation rather than as a direct rescue experiment. Either stronger causal evidence is needed, or the wording should be softened.

Finally, the term "rescue" is sometimes overstated. For example, in the Pten/Rictor double knockout condition, some measurements fall below WT levels rather than simply returning to normal. In these cases, "suppressed" or "partially reversed" would be more accurate than "rescued."

Overall, this is a solid study suitable for eNeuro, but revision is needed to better calibrate the claims and strengthen the interpretation.

Reviewer #2:

This manuscript addresses an important question regarding how PTEN regulates microtubule (MT) dynamics through distinct mTOR signaling pathways in peripheral sensory neurons. The study is well-motivated, and the identification of mTORC2 as a key mediator of MT polymerization at the growth cone is potentially impactful. The experimental approach is appropriate, and the automated tracking pipeline is a strength of the study. However, several issues related to clarity, terminology, interpretation, and completeness of reporting should be addressed.

A central issue is the interpretation and consistency of the PTEN-KO phenotype. The authors state that PTEN-KO increases MT polymerization and neuronal hypertrophy, but this is not clearly or consistently defined. It appears that "hypertrophy" includes increases in neurite outgrowth, soma size, and distal axonal growth; however, these represent distinct biological outcomes. The manuscript currently combines measurements of MT polymerization, axonal elongation, neurite complexity (via Sholl analysis), and soma size without clearly distinguishing between them. The authors should explicitly define what they mean by hypertrophy and align all measurements and interpretations accordingly. Related to this, the manuscript refers interchangeably to axonal growth, neurite outgrowth, and branching, which are not equivalent metrics. Sholl analysis reflects neurite complexity rather than strictly axonal elongation, and these distinctions should be clarified throughout.

The use of the term "rescue" is also problematic. In several instances, including the description of mTORC2 suppression effects, this term implies a beneficial or corrective outcome. However, based on the data, mTORC2 suppression appears to reverse or attenuate PTEN-KO-induced changes rather than restore a pathological state. This wording should be revised for clarity and accuracy.

Abstract/Significance/Introduction

Lines 31-33: The authors state that suppression of mTORC2, but not mTORC1, rescues PTEN-KO-driven accelerated MT polymerization and neuronal hypertrophy. This should be clarified. Are the authors concluding that PTEN-KO drives both increased MT polymerization and neuronal hypertrophy? If so, this should be stated clearly and consistently. The authors should also specify whether these effects are restricted to the axon terminal/growth cone and not the axon shaft.

Line 46: The use of "rescue" should be reconsidered, as noted above.

Lines 83-87: PTEN-KO is described as driving neuronal hypertrophy, but this term is not defined. The authors should specify which measurements (e.g., soma size, neurite length, distal growth) constitute hypertrophy.

Lines 88-92: The authors suggest PTEN differentially regulates MT polymerization across subcellular compartments. It would be helpful to discuss whether these findings are expected to extend to CNS neurons or are specific to peripheral sensory neurons.

Discussion

Lines 406-430: This section primarily summarizes prior studies. The Discussion would benefit from clearer interpretation of the authors' findings and how they advance understanding of PTEN/mTOR signaling, MT dynamics, and axonal growth.

Methods/Reporting

Line 97: Missing university information.

Line 98: Missing author/laboratory information.

Line 99: Missing university information.

Line 153: Define the acronym "PLL."

Line 162: The authors should report transfection efficiency when using calcium phosphate precipitation.

Line 193: Please specify the type/model of confocal microscope used for data acquisition.

Statistical analyses are not sufficiently described. The authors should clearly state which tests were used for each dataset, whether assumptions were assessed, how multiple comparisons were handled, and how sample sizes and replicates were defined.

Results

Line 207: The rationale for using EB3-GFP is not explained. The authors should clarify why labeling MT plus ends is an appropriate readout for MT polymerization and how this relates to the study's hypothesis.

Line 248: The sentence describing vinblastine effects is incomplete ("from" value missing). The paragraph is also unclear in its current form. The authors should more clearly present how vinblastine alters MT polymerization and how this validates the automated pipeline.

Lines 255-263: This paragraph may be better placed within the subsequent section on PTEN-KO effects or shortened to a brief validation statement.

Lines 265-280: There is ambiguity in how growth outcomes are described. The authors report increased MT polymerization, but also present Sholl analysis and soma size data. It is unclear whether they are referring to axonal elongation, neurite complexity, soma growth, or all of these. These outcomes should be clearly distinguished and described consistently.

Line 343: There appears to be a switch from RAPTOR (PtRap-DKO) to RICTOR (PtRic-KO) that may be incorrect. Please clarify.

Generally, there is a section describing PtRap-DKO, but the results supporting the role of Rictor (PtRic-DKO) in PTEN-mediated axonal growth and MT regulation are not in the Results section and these should be clearly emphasized, as this is central to the manuscript's main conclusion.

Software/Pipeline

The automated MT tracking pipeline using arivis4D is a strength of the study and is appropriately validated against manual tracking and pharmacological perturbation. However, reproducibility is unclear. Several steps rely on user-defined parameters (e.g., filtering radius, segmentation thresholds), but these are not fully specified. It is also not clear whether the pipeline or analysis scripts are publicly available. The authors should provide sufficient detail to enable replication and clarify accessibility.

## Author Response

Synthesis Statement for Author (Required):

Both reviewers agree this is a well-conceived, technically solid study that uses an appropriate conditional co-deletion strategy and a well-validated EB3 tracking pipeline to establish mTORC2, rather than mTORC1, as the key mediator of PTEN-regulated microtubule polymerization and growth in adult DRG neurons. However, both raise substantive concerns that the claims are overstated relative to the data-particularly the use of "rescue," the translational/therapeutic framing in the absence of in vivo or injury-model evidence, and the still-speculative downstream link between mTORC2 and microtubule regulation (e.g., AKT/GSK3β/CRMP2). Revisions should focus on tempering interpretation, clearly defining and disambiguating outcome measures (hypertrophy vs. neurite outgrowth vs. axonal elongation vs. complexity), strengthening causal evidence for the microtubule-dependence of PTEN-driven growth, and improving reporting of statistics, methodological details, and pipeline reproducibility. • We thank the editor and reviewers for the opportunity to resubmit our manuscript to eNeuro. We have carefully reviewed the reviewer comments and have addressed all concerns in the revised manuscript. In particular, we have carefully noted and clarified all uses of the word "rescue" in the manuscript to align more closely to the data and avoided over-interpretation of our findings and all outcome measures are now explicitly defined throughout. Additionally, we have removed and modified language regarding the therapeutic framing as we did not use an injury model and explicitly include this in our discussion of limitations. The discussion section has been extensively revised to address concerns regarding readability and our conclusions are more nuanced. Lastly, we have improved our methods section for increased rigor and reproducibility including adding a statistical analysis subsection and sharing our github (https://github.com/smevans19/puncta-tracking-arivis. ) for the pipeline tracking code. All of the key changes are highlighted in our tracked revision using highlighted text.

Reviewers' comments:

Reviewer #1:

The manuscript is well written, logically organized, and technically solid. The authors clearly define the question and use appropriate experiments to show that PTEN regulates growth cone microtubule polymerization primarily through mTORC2 rather than mTORC1 in adult DRG neurons. The conditional co-deletion strategy is appropriate, and the automated EB3 tracking pipeline is carefully validated, which strengthens confidence in the results. • We thank the reviewer for their encouraging comments, and appreciate their careful review of our manuscript. We appreciate the following comments and have revised our manuscript in response, which has made for an overall stronger and clearer manuscript.

My main concern is that the significance and translational implications are overstated. The study is entirely based on in vitro DRG cultures and does not include an injury model, in vivo regeneration, or functional recovery. Therefore, statements describing microtubules as a strong therapeutic target for peripheral regeneration should be toned down. • We appreciate this feedback and have restated our conclusions to reflect a more conservative framework, "Finally, we show the relationship between MT dynamics and overall neuronal morphology, and highlight the MT cytoskeleton as a vital component of peripheral nerve regeneration that may have future clinical applications." (lines 100-102). • We agree that the study results would be strengthened by an in vivo regeneration model, and have added mention of this to our discussion section : "An important future experiment would be to study the effects of PtRap-DKO in an in vivo model of peripheral nerve injury and regeneration, since our in vitro culture system may have obscured the long-term effects of Raptor on neuronal regeneration." (lines 504-507). Our lab is currently investigating promotion of regeneration in an in vivo model of chronic neuropathy, and will describe this work in a future manuscript. • We have additionally revised the discussion section of the current manuscript to reflect a more conservative interpretation of the data. We have changed: "MTs are also especially promising as a therapeutic target for neuronal regeneration because there already exist a wide variety of FDA-approved microtubule-binding drugs, both stabilizing and destabilizing. These drugs have been well-characterized and are highly effective and specific in binding to MTs (Fanale et al. 2015). Here, we find Pten regulates MT cytoskeletal dynamics in the axonal growth cone, but not the shaft; and that this regulation occurs through mTORC2 and not mTORC1. These results support a growth cone-specific pathway of MT polymerization regulation via mTORC2-Akt-GSK3beta." to "Here, we find Pten regulates MT cytoskeletal dynamics in the axonal growth cone, but not the shaft; and that this regulation occurs through mTORC2 and not mTORC1. Identifying a region-specific correlation between MT polymerization rates and axonal outgrowth is a novel finding, and supports a growth cone-specific pathway of MT polymerization regulation via mTORC2." (lines 558-563).

A second concern is that the mechanistic pathway remains incomplete. While the data support that mTORC2, but not mTORC1, is required for PTEN-mediated regulation of microtubule dynamics, the downstream link between mTORC2 and microtubule regulation is not directly tested. Molecules such as AKT, GSK3β, and CRMP2 are only discussed speculatively, so the conclusions should be more cautious. • We agree with the reviewer that direct study of the link between mTORC2 and microtubule regulation would strengthen our results. We have revised our discussion to highlight how our data fits into what is already known about the broader signaling landscape downstream of mTORC2, and more clearly indicate that the exact intermediary mechanism remains unresolved based on our observations, and warrants future study. We have added: "However, as our study did not directly observe signaling molecules in between mTORC2 and MT polymerization, the exact intermediary mechanism remains unresolved, and warrants future study." (lines 529-531).

In addition, the claim that PTEN-driven neurite growth depends on microtubule activation is still largely correlative. Vinblastine is used only for pipeline validation rather than as a direct rescue experiment. Either stronger causal evidence is needed, or the wording should be softened. • We have softened the wording to clarify that the relationship between neurite growth and microtubule activation is strongly correlative, but not necessarily causative. For example, we have added: "These increases in Sholl of intersections, Sholl of length, and soma size reflect a morphological phenotype of substantially increased soma size and neurite length compared to WT control neurons, and are correlated with observed changes in cytoskeletal dynamics." to our results section (lines 352-355), and "However, as our study did not directly observe signaling molecules in between mTORC2 and MT polymerization, the exact intermediary mechanism remains unresolved, and warrants future study." to the discussion (lines 526-528). We have also softened existing wording in several places, such as changing "Therefore, we conclude that mTORC2 is necessary for Pten-KO's effects..." to "Therefore, we conclude that mTORC2 mediates Pten-KO's effects..." (lines 402-404).

Finally, the term "rescue" is sometimes overstated. For example, in the Pten/Rictor double knockout condition, some measurements fall below WT levels rather than simply returning to normal. In these cases, "suppressed" or "partially reversed" would be more accurate than "rescued." • We agree that the term "rescue" may be inappropriate in the context of this study. We have revised instances of the "rescue" throughout the entire manuscript to wording that more accurately reflects the data as the reviewer suggests (see highlighted text throughout).

Overall, this is a solid study suitable for eNeuro, but revision is needed to better calibrate the claims and strengthen the interpretation. • We thank the reviewer for their careful review of our manuscript and thoughtful feedback.

Reviewer #2:

This manuscript addresses an important question regarding how PTEN regulates microtubule (MT) dynamics through distinct mTOR signaling pathways in peripheral sensory neurons. The study is well-motivated, and the identification of mTORC2 as a key mediator of MT polymerization at the growth cone is potentially impactful. The experimental approach is appropriate, and the automated tracking pipeline is a strength of the study. However, several issues related to clarity, terminology, interpretation, and completeness of reporting should be addressed. • We appreciate that the reviewer sees value in our data, and thank them for the careful review of our manuscript. We have revised the manuscript, particularly the discussion section, to reflect their feedback regarding clarity and interpretation of data.

A central issue is the interpretation and consistency of the PTEN-KO phenotype. The authors state that PTEN-KO increases MT polymerization and neuronal hypertrophy, but this is not clearly or consistently defined. It appears that "hypertrophy" includes increases in neurite outgrowth, soma size, and distal axonal growth; however, these represent distinct biological outcomes. The manuscript currently combines measurements of MT polymerization, axonal elongation, neurite complexity (via Sholl analysis), and soma size without clearly distinguishing between them. The authors should explicitly define what they mean by hypertrophy and align all measurements and interpretations accordingly. Related to this, the manuscript refers interchangeably to axonal growth, neurite outgrowth, and branching, which are not equivalent metrics. Sholl analysis reflects neurite complexity rather than strictly axonal elongation, and these distinctions should be clarified throughout. • We have gone through the manuscript and replaced uses of the term "hypertrophy" with more specific terminology, to more clearly communicate which specific physiological/morphological feature is being measured. We agree with the reviewer that more clearly delineating different measurements has improved manuscript quality and readability. Specifically, we have made significant edits in the results section. • We have changed "outgrowth" in the section title to more specific phrasing, "soma size, axon length, and neurite branching" (line 333). Similarly, we have changed "Fixed cell images were digitally reconstructed, and Sholl analysis was performed to quantify neurite outgrowth" to "Fixed cell images were digitally reconstructed, and Sholl analysis was performed to quantify changes in neurite morphology, including length and branching complexity" (lines 342-344). • We have also added "Pten-KO increases the average intersections and length specifically at radii further from the soma, which describes a pattern of neurite elongation, rather than increased branching complexity. These morphological changes are described in more detail later in this study (fig 4). (lines 347-350). • We have changed "overall neuronal growth" to "neuronal morphology" (line 403), "outgrowth" to "neurite extension and branching" (line 381), "rescue Pten-KO-driven hypertrophy at the cellular level" to "reverse the morphological changes observed in Pten-KO neurons" (line 395), and "neuronal growth" to "cytoskeletal dynamics and neuronal morphology" (line 403).

The use of the term "rescue" is also problematic. In several instances, including the description of mTORC2 suppression effects, this term implies a beneficial or corrective outcome. However, based on the data, mTORC2 suppression appears to reverse or attenuate PTEN-KO-induced changes rather than restore a pathological state. This wording should be revised for clarity and accuracy. • Thank you for this feedback. We agree that the term "rescue" is not appropriate for these results, and have revised the wording throughout the manuscript to more accurately reflect the observed impact of mTORC2 suppression. Please see the revised text below: mTORC2 loss of function reverses Pten-KO-mediated hypertrophy in peripheral sensory neurons We next investigated the effects of the mTORC2 signaling pathway. We cultured DRG neurons from Ptenfl/fl x Rictorfl/fl mice, transfected with a Cre-containing plasmid to generate Pten-KO x Rictor-KO (PtRic-DKO) cells or a control plasmid to generate WT neurons, and live-imaged to quantify MT polymerization rates as previously described (fig 3A-B). Co-deletion of Rictor appeared to fully reverse the accelerated MT polymerization rates in Pten-KO neurons: PtRic-DKO cells demonstrated MT velocity rates similar to WT levels, significantly decreased from Pten-KO levels (PtRic-DKO = 0.08287{plus minus}0.01133um/s, WT vs. PtRic-DKO p=0.9442, Pten-KO vs. PtRic-DKO p=0.0005) (fig 3C). Similarly, Rictor-KO appeared to reverse the morphological changes observed in Pten-KO neurons. We fixed and stained cells to quantify cellular outgrowth (fig 3D) as previously described. We found PtRic-DKO cells had similar Sholl of intersections and length compared to WT cells, decreased from that of Pten-KO cells (fig 3E-F). We also found that Rictor-KO reduces soma size (PtRic-DKO = 207.0{plus minus}61.06um2, WT vs PtRic-DKO p<0.0001, Pten-KO vs PtRic-DKO p=0.0006) (fig 3G) and total neurite length (PtRic-DKO = 430.8{plus minus}342.2um, WT vs PtRic-DKO p=0.0506, Pten-KO vs PtRic-DKO p<0.0001) (fig 3H) from elevated Pten-KO levels to near-WT levels. Therefore, we conclude that mTORC2 mediates Pten-KO's effects on cytoskeletal dynamics and neuronal morphology in peripheral sensory neurons. (lines 385-404).

Abstract/Significance/Introduction Lines 31-33: The authors state that suppression of mTORC2, but not mTORC1, rescues PTEN-KO-driven accelerated MT polymerization and neuronal hypertrophy. This should be clarified. Are the authors concluding that PTEN-KO drives both increased MT polymerization and neuronal hypertrophy? If so, this should be stated clearly and consistently. The authors should also specify whether these effects are restricted to the axon terminal/growth cone and not the axon shaft. • We have edited this sentence for improved clarity. We have changed: "We show that suppression of mTORC2, but not mTORC1, is sufficient to rescue Pten-KO-driven accelerated MT polymerization and neuronal hypertrophy. These results are specific to the axonal growth cone, and MT dynamics in the proximal axon shaft are not impacted by Pten-KO." to "We use Pten-KO to increase MT polymerization and neuronal outgrowth, and then show that suppression of mTORC2, but not mTORC1, is sufficient to reduce the accelerated MT polymerization and neuronal hypertrophy to wild-type levels. These results are specific to the axonal growth cone, and MT dynamics in the proximal axon shaft are not impacted by Pten-KO, mTORC1 suppression, or mTORC2 suppression." (lines 33-35).

Line 46: The use of "rescue" should be reconsidered, as noted above. • We agree that the terminology should be clarified. We have revised the wording from "This is rescued by co-deletion of mTORC2, but not mTORC1. We also found mTORC2 suppression rescues distal axonal elongation from Pten-KO..." to "We also find that loss of mTORC2 inhibits elongation of the distal axon in the context of Pten-KO, suggesting that the growth cone-specific effects of Pten/mTORC2 on the MT cytoskeleton may regulate axonal outgrowth." (lines 47-50).

Lines 83-87: PTEN-KO is described as driving neuronal hypertrophy, but this term is not defined. The authors should specify which measurements. • In this section, we have revised the wording from "neuronal hypertrophy" to "neurite extension, somal hypertrophy, and accelerated MT polymerization". We have made additional revisions throughout the manuscript to change most instances of "hypertrophy" to more specific language.

Lines 88-92: The authors suggest PTEN differentially regulates MT polymerization across subcellular compartments. It would be helpful to discuss whether these findings are expected to extend to CNS neurons or are specific to peripheral sensory neurons. • We have expanded our discussion section to place the novelty of this finding in the context of what is broadly known about MT polymerization in difference subcellular compartments. "To our knowledge, there are no previous studies that have examined Pten-mediated MT polymerization changes across axon compartments in the CNS. The MT cytoskeleton appears to have differential patterns of polymerization in axons versus dendrites in cultured hippocampal neurons (Kollins et al. 2009), but whether MT polymerization varies within the different regions of the axon, or whether these distinct processes are regulated by Pten is unknown." (lines 551-556).

Discussion Lines 406-430: This section primarily summarizes prior studies. The Discussion would benefit from clearer interpretation of the authors' findings and how they advance understanding of PTEN/mTOR signaling, MT dynamics, and axonal growth. • We agree that this section is improved by clearer interpretation of our findings and how our findings build upon and relate to existing literature. We have heavily revised this section. The revised text condenses explanation of past studies, and focuses more on explaining how our results expand understanding of this pathway. The new text reads: "First, we found knocking out Raptor (mTORC1) reduced distal neurite outgrowth, but did not reverse the effects of Pten-KO on the MT cytoskeleton with regard to polymerization velocity (fig 2C). Knocking out Raptor also did not significantly impact soma hypertrophy compared to Pten-KO neurons (fig 2G). This was a surprising finding, as previous studies have shown that both conditional co-deletion of mTORC1 in vivo, as well as direct pharmacological inhibition of mTORC1 by rapamycin, were sufficient to rescue Pten-loss mediated phenotypes of neuronal hypertrophy and aberrant migration of hippocampal dentate gyrus neurons in neonatal mice (Getz et al. 2016, Tariq et al. 2022). Likewise, in human neural organoids, either mTORC1-KO or mTORC2-KO independently was sufficient to rescue hypertrophy and electrical hyperactivity caused by Pten-KO (Dhaliwal et al. 2024).While these earlier findings support a role for mTORC1 in regulation of neuronal hypertrophy, they may be context-specific: neonatal mice and brain organoids model early CNS development, not adult peripheral nerve regeneration, as in our study. Additionally, mTORC1 is thought to regulate cell growth primarily through regulating protein translation (Saxton and Sabatini 2017, Laplante and Sabatini 2009, Takei and Nawa 2014). This process takes time and may therefore not have an immediate observable impact on cytoskeleton dynamics over the acute time periods in our study (2-4 days). Similarly, our in vitro experimental did not support observations over the much longer timecourse of previous in vivo models (Getz et al. 2016, Tariq et al. 2022). An important future experiment would be to study the effects of PtRap-DKO in an in vivo model of peripheral nerve injury and regeneration, since our in vitro culture system may have obscured the long-term effects of Raptor on neuronal regeneration and could not test functional outcomes". (lines 486-507).

Methods/Reporting Line 97: Missing university information. • The university affiliation of one of our authors was removed from the initial submission, per eNeuro's anonymity/double-blind guidelines.

Line 98: Missing author/laboratory information. • This identifying information for one of our authors was removed from the initial submission, per eNeuro's anonymity/double-blind guidelines.

Line 99: Missing university information. • The university affiliation of one of our authors was removed from the initial submission, per eNeuro's anonymity/double-blind guidelines.

Line 153: Define the acronym "PLL." • We have changed all uses of the acronym to the full name (poly-L-lysine).

Line 162: The authors should report transfection efficiency when using calcium phosphate precipitation. • We have updated the methods section to report the transfection efficiency: "This yielded a dual transfection efficiency of approximately 10%, allowing for imaging of isolated neurons in sparsely labeled cultures." (lines 179-181).

Line 193: Please specify the type/model of confocal microscope used for data acquisition. • We have added the following: "Fixed cells were imaged using a Zeiss LSM880 confocal microscope using a 20X objective." (lines 209-210).

Statistical analyses are not sufficiently described. The authors should clearly state which tests were used for each dataset, whether assumptions were assessed, how multiple comparisons were handled, and how sample sizes and replicates were defined. • Thank you for this feedback. We have added a description of our statistical analyses to the Methods section:

Statistical analysis. All statistical analyses and graphical representations of the data were generated in Graphpad Prism 10, unless otherwise noted. For all microtubule data, all microtubule velocities across all timepoints for a single cell were averaged to generate a single data point, and reported n values are the number of cells. Reported mean and standard deviation are the mean and standard deviation across all cells per condition. For all morphological (soma size, Sholl analysis) data, n values are a single cell. For both microtubule and morphological experiments, data from at least three distinct culture sets (in other words, three individual animals) was reported for each genetic condition, to compensate for potential individual variability. Minimum sample size was determined by performing a power analysis based on prior work from our lab (citations removed for anonymity).

Exact statistical tests used are reported in relevant figure legends. We used Welch's t-test to compare manual vs. automated tracking and did not perform multiple comparisons (fig 1). To compare MT velocities, soma size, sum of intersections, and sum of length across genetic conditions, we first used Welch and Brown-Forsythe one-way ANOVA tests to determine significance, and then used Dunnett's T3 multiple comparisons test to examine specific inter-condition comparisons (fig. 2-4, tables 1-6). To compare Sholl analysis results across genetic conditions, we performed a two-way ANOVA, with radius and condition as factors. We then used Tukey's multiple comparisons tests to examine differences across genetic conditions (fig. 2-3). (lines 221-240).

Results Line 207: The rationale for using EB3-GFP is not explained. The authors should clarify why labeling MT plus ends is an appropriate readout for MT polymerization and how this relates to the study's hypothesis. • We have added a brief explanation of microtubule structure to the introduction: "Microtubules are polar, self-assembling polymers, composed of a coiled chain of alternating alpha- and beta-tubulin monomers. Microtubules polymerize and de-polymerize from the "plus" end, which has an exposed beta-tubulin subunit and serves as an important binding site for regulatory proteins, such as end-binding protein 3 (EB3)." (lines 78-82). • We have also added a brief explanation to the results section: "EB3 (end-binding protein 3) binds to the actively-polymerizing plus-ends, so EB3-GFP labeling provides a visual readout of MT polymerization." (lines 273-275) Line 248: The sentence describing vinblastine effects is incomplete ("from" value missing). The paragraph is also unclear in its current form. The authors should more clearly present how vinblastine alters MT polymerization and how this validates the automated pipeline. o We have added text and a relevant citation to this paragraph to more clearly present how vinblastine alters MT polymerization. "After validating pipeline accuracy to manual tracking, we then sought to validate the pipeline's ability to detect potential differences in MT velocity across experimental conditions, using both pharmacological and genetic manipulations. First, we intentionally reduced MT velocity by acute in vitro treatment with vinblastine, a chemotherapy drug which is known to decrease MT polymerization velocity by binding directly to the beta-tubulin subunit of tubulin dimers, causing them to aggregate into non-functional spiral polymers instead of forming normal MTs (Toso et al. 1993, Dhamodharan et al. 1995, Gigant et al. 2005). A previous study in neonatal hippocampal neuronal cultures found acute vinblastine treatment significantly reduced MT polymerization velocity in vitro (Getz et al. 2022). We treated DRG cell cultures with 2nM vinblastine and imaged them 30-120min post-treatment." (lines 305-315).

Lines 255-263: This paragraph may be better placed within the subsequent section on PTEN-KO effects or shortened to a brief validation statement. • We have shortened this paragraph in order to more clearly present it in the context of pipeline validation. "Next, we used a genetic manipulation to intentionally increase MT polymerization rates. Previous work (Getz et al. 2022) has found that Pten-KO significantly increased MT polymerization rates in the axonal growth cone of embryonic hippocampal neurons. After culturing, we generated Pten-KO neurons, and found no significant difference between velocities generated by manual and automated puncta-tracking (0.1041{plus minus}0.03067um/s vs 0.1036 {plus minus}0.01488um/s, p =0.9631, fig 1E). (lines 322-328).

Lines 265-280: There is ambiguity in how growth outcomes are described. The authors report increased MT polymerization, but also present Sholl analysis and soma size data. It is unclear whether they are referring to axonal elongation, neurite complexity, soma growth, or all of these. These outcomes should be clearly distinguished and described consistently. • Thank you for this feedback. We have significantly edited this section in order to clarify what specific growth outcomes our data shows. We have changed "outgrowth" in the section title to more specific phrasing, "soma size, axon length, and neurite branching". • Similarly, we have changed "Fixed cell images were digitally reconstructed, and Sholl analysis was performed to quantify neurite outgrowth" to "Fixed cell images were digitally reconstructed, and Sholl analysis was performed to quantify changes in neurite morphology, including length and branching complexity". • We have also added "Pten-KO increases the average intersections and length specifically at radii further from the soma, which describes a pattern of neurite elongation, rather than increased branching complexity. These morphological changes are described in more detail later in this study (fig 4). Finally, in addition to changes in neurite morphology, Pten-KO significantly increases soma size over WT neurons (WT = 274.1{plus minus}86.26um2, Pten-KO = 358.1{plus minus}138.7um2, p = 0.0031) (fig. 2G). These increases in Sholl of intersections, Sholl of length, and soma size reflect a morphological phenotype of substantially increased soma size and neurite length compared to WT control neurons, and are correlated with the increased MT velocity we observed in the same cells." (lines 348-356). • We have similarly edited subsequent results sections to more clearly distinguish growth outcomes and use more precise language.

Line 343: There appears to be a switch from RAPTOR (PtRap-DKO) to RICTOR (PtRic-KO) that may be incorrect. Please clarify. • We realize this sentence may cause confusion for the reader, since this PtRic-DKO finding is counterintuitive relative to our other findings. We have revised our wording and added additional discussion to explicitly clarify that this finding is in contrast to PtRic-KO's suppressive effects in other contexts. We have changed: "Specifically, MT polymerization velocity in WT cells is non-significantly increased in the proximal axon shaft, with no significant difference between WT and Pten-KO velocities (fig. 4B, tables 1-2). Additionally, PtRic-KO cells have significantly increased polymerization velocity in the proximal axon shaft compared to the growth cone (fig. 4B, tables 1-2), implying region-specific differences in polymerization or regulation." to "Specifically, MT polymerization velocity in WT cells is non-significantly increased in the proximal axon shaft compared to the growth cone, with no significant difference between WT and Pten-KO velocities in the proximal axon shaft (fig. 4B, tables 1-2). Interestingly, there was no significant difference between Pten-KO and PtRic-DKO MT polymerization velocities within the axon shaft (fig. 4B, tables 1-2). This is in apparent contrast to our findings in the growth cone, where PtRic-DKO significantly reduced polymerization rates from Pten-KO, implying region-specific differences in polymerization or regulation." (lines 423-440). • We have also added content to the discussion section for clarifiation: "Finally, PtRic-DKO neurons demonstrated significant decreases compared to Pten-KO neurons in sum of intersections and length at all distances, proximal and distal. This contrasts with the observed elevated MT polymerization rates in PtRic-DKO axon shafts, and suggests that MT-driven mechanisms of axon growth may be region-specific." (lines 444-448).

Generally, there is a section describing PtRap-DKO, but the results supporting the role of Rictor (PtRic-DKO) in PTEN-mediated axonal growth and MT regulation are not in the Results section and these should be clearly emphasized, as this is central to the manuscript's main conclusion. • Thank you for alerting us to this potential miscommunication. We have carefully reviewed the manuscript and describe the role of Rictor in the Results section (lines 386-408), and discussed its importance throughout the discussion sections, particularly in the paragraph beginning at line 509, in order to emphasize its importance in our manuscript's main conclusion. "In the present study, co-deletion of Rictor (mTORC2) reversed the increased neurite length, neurite branching, and soma size of PTEN-KO (fig 3). This aligns with a previous study showing mTORC2-KO rescued morphological effects of Pten-KO in dentate gyrus neurons, particularly effects distal to the cell body (e.g. arborization and branching), but not more proximal effects (soma size or number of primary dendrites) (Cullen et al. 2023). We also observed that mTORC2 altered growth cone MT dynamics from Pten-KO, significantly reducing MT polymerization rates. Existing literature has established a strong link between mTORC2 and the actin cytoskeleton (Laplante and Sabatini 2009, Angliker and Rüegg 2013, Huang et al. 2013), but the effects of mTORC2 on the microtubule cytoskeleton have not been well-studied. mTORC2 may affect the MT cytoskeleton through either of two potential downstream factors: CRMP2, which binds to tubulin heterodimers, promotes polymerization, and plays a role in axonal growth cone guidance (Inagaki et al. 2001, Fukata et al. 2002); and MAP1B, which binds to dynamic (tyrosinated) microtubules and thus regulates MT polymerization dynamics (Kawauchi and Hoshino 2003, Bouquet et al. 2004, Tymanskyj et al. 2012). Activity in this pathway due to Pten-KO would, in theory, cause an increase in stable, detyrosinated MTs, and an increase in CRMP2 expression (which would then promote polymerization). Indeed, Pten-KO can increase levels of stable (detyrosinated) microtubules, and Pten-KO driven hypertrophy can be reversed through destabilizing (tyrosinating) the MTs directly (Kath et al. 2018). Both the CRMP2- and MAP1B-driven polymerization effects would theoretically be reversed by Rictor (mTORC2) knockout, which could explain our observed decrease in MT polymerization rates. However, as our study did not directly observe signaling molecules in between mTORC2 and MT polymerization, the exact intermediary mechanism remains unresolved, and warrants future study." Software/Pipeline The automated MT tracking pipeline using arivis4D is a strength of the study and is appropriately validated against manual tracking and pharmacological perturbation. However, reproducibility is unclear. Several steps rely on user-defined parameters (e.g., filtering radius, segmentation thresholds), but these are not fully specified. It is also not clear whether the pipeline or analysis scripts are publicly available. The authors should provide sufficient detail to enable replication and clarify accessibility. • We agree reproducibility and rigor are very important. We have made our code publicly available at: https://github.com/smevans19/puncta-tracking-arivis. • We have also added a section to the methods describing our code, including the github repository link, and details regarding the precise user-defined parameters. "Automated puncta-tracking. Live-imaged cells were processed using an automated puncta-tracking pipeline we developed in the software arivis4D, from Zeiss. The full pipeline and documentation is publicly available at https://github.com/smevans19/puncta-tracking-arivis. In brief, the pipeline consists of two broad steps: segmenting (puncta identification) and tracking (identifying puncta movement over time).

Segmenting was performed by performing bilateral denoising, then using arivis' automated "blob finder" algorithm to identify fluorescent puncta (i.e. labeled MT plus-ends) according to a user-determined probability threshold. Finally, we applied a size-based exclusion filter to reduce segments below a user-determined area threshold, reducing false positives and background noise detection.

Tracking was performed by importing the segments generated during the previous step, then using arivis' "Tracking" algorithm to link segment centroids across consecutive frames, with linear regression-based trajectories. One important user-determined parameter was the max distance traveled across frames, which was calculated by measuring the distance traveled by a visibly fast-moving punctum across 2 timepoints, for 10 sets of timepoints distributed across the imageset, then averaging these distances and rounding to the nearest 25nm. Weighting was biased towards motion, rather than geometric feature, since MTs follow consistently linear paths (motion), but the appearance of a labeled MT (feature) can vary widely across frames due to imaging variability.

Finally, track data was exported, and used to calculate MT polymerization velocity for each track. Track velocity was then averaged, and average velocity per cell is reported. More details and documentation for the arivis segmenting/tracking pipeline can be found at https://github.com/smevans19/puncta-tracking-arivis." (lines 242-266).

We sincerely thank the reviewer for the helpful suggestions and feel that our paper has been greatly improved due to their efforts.
