# Supplementary material for: PTEN Regulates Microtubule Polymerization via mTORC2, But Not mTORC1, in Peripheral Sensory Neurons
Source: eNeuro. 2026 Sep 8;13(9):ENEURO.0116-26.2026. doi: 10.1523/ENEURO.0116-26.2026 (PMC13640863; doi:10.1523/ENEURO.0116-26.2026)
Supplement: Supplementary file 1 [file eneuro-13-ENEURO.0116-26.2026-s004.docx]

| Region and genetic condition | Mean (um/s) | Std. Dev. (um/s) | n (cells) |
| --- | --- | --- | --- |
| Distal WT | 0.08024 | 0.01494 | 33 |
| Distal *Pten-*KO | 0.0968 | 0.01883 | 42 |
| Distal *Pten-*KO x *Raptor-*KO | 0.1039 | 0.02019 | 35 |
| Distal *Pten-*KO x *Rictor-*KO | 0.08287 | 0.01133 | 51 |
| Proximal WT | 0.1011 | 0.01571 | 20 |
| Proximal *Pten-*KO | 0.1114 | 0.02486 | 20 |
| Proximal *Pten-*KO x *Raptor-*KO | 0.1111 | 0.01527 | 20 |
| Proximal *Pten-*KO x *Rictor-*KO | 0.1022 | 0.02051 | 20 |
