# Supplementary material for: PTEN Regulates Microtubule Polymerization via mTORC2, But Not mTORC1, in Peripheral Sensory Neurons
Source: eNeuro. 2026 Sep 8;13(9):ENEURO.0116-26.2026. doi: 10.1523/ENEURO.0116-26.2026 (PMC13640863; doi:10.1523/ENEURO.0116-26.2026)
Supplement: Supplementary file 2 [file eneuro-13-ENEURO.0116-26.2026-s005.docx]

| Dunnett's T3 multiple comparisons test | Adjusted P Value |
| --- | --- |
| **Within-growth cone comparisons** |  |
| **Gc WT (38) vs. Gc Pten-KO (42)** | **0.0017** |
| Gc Pten-KO (42) vs. Gc PtRap-DKO (21) | 0.2614 |
| **Gc WT (38) vs. Gc PtRap-DKO (21)** | **<0.0001** |
| Gc WT (38) vs. Gc PtRic-DKO (51) | >0.9999 |
| **Gc Pten-KO (42) vs. Gc PtRic-DKO (51)** | **0.0023** |
|  |  |
| **Within-shaft comparisons** |  |
| Shaft WT (20) vs. Shaft Pten-KO (20) | 0.9486 |
| Shaft WT (20) vs. Shaft PtRap-DKO (20) | 0.6735 |
| Shaft WT (20) vs. Shaft PtRic-DKO (20) | >0.9999 |
|  |  |
| Shaft Pten-KO (20) vs. Shaft PtRap-DKO (20) | >0.9999 |
| Shaft Pten-KO (20) vs. Shaft PtRic-DKO (20) | 0.9952 |
|  |  |
| **Cross-region comparisons** |  |
| **Gc WT (38) vs. Shaft WT (20)** | **0.0007** |
| Gc Pten-KO (42) vs. Shaft Pten-KO (20) | 0.4691 |
| **Gc PtRic-DKO (51) vs. Shaft PtRic-DKO (20)** | **0.0143** |
| Gc PtRap-DKO (21) vs. Shaft PtRap-DKO (20) | >0.9999 |
