# Supplementary material for: PTEN Regulates Microtubule Polymerization via mTORC2, But Not mTORC1, in Peripheral Sensory Neurons
Source: eNeuro. 2026 Sep 8;13(9):ENEURO.0116-26.2026. doi: 10.1523/ENEURO.0116-26.2026 (PMC13640863; doi:10.1523/ENEURO.0116-26.2026)
Supplement: Supplementary file 3 [file eneuro-13-ENEURO.0116-26.2026-s006.docx]

|  | Mean (sum of intersections) | Std. Dev. | n (cells) |
| --- | --- | --- | --- |
| Proximal WT | 43.04 | 26.12 | 48 |
| Proximal *Pten-*KO | 53.08 | 20.13 | 51 |
| Proximal *Pten-*KO x *Raptor-*KO | 61.87 | 36.22 | 23 |
| Proximal *Pten-*KO x *Rictor-*KO | 30 | 23.47 | 50 |
| Distal WT | 9.479 | 26.11 | 48 |
| Distal *Pten-*KO | 44.63 | 47.06 | 51 |
| Distal *Pten-*KO x *Raptor-*KO | 13.78 | 34 | 23 |
| Distal *Pten-*KO x *Rictor-*KO | 1.36 | 4.776 | 50 |
