# Supplementary material for: PTEN Regulates Microtubule Polymerization via mTORC2, But Not mTORC1, in Peripheral Sensory Neurons
Source: eNeuro. 2026 Sep 8;13(9):ENEURO.0116-26.2026. doi: 10.1523/ENEURO.0116-26.2026 (PMC13640863; doi:10.1523/ENEURO.0116-26.2026)
Supplement: Supplementary file 4 [file eneuro-13-ENEURO.0116-26.2026-s007.docx]

| Dunnett's T3 multiple comparisons test | Adjusted P value |
| --- | --- |
| Proximal intersections |  |
| WT (48) vs. Pten-KO (51) | 0.1938 |
| WT (48) vs. PtRap-DKO (23) | 0.1742 |
| WT (48) vs. PtRic-DKO (50) | 0.0633 |
| Pten-KO (51) vs. PtRap-DKO (23) | 0.85 |
| **Pten-KO (51) vs. PtRic-DKO (50)** | **<0.0001** |
| Distal intersections |  |
| **WT (48) vs. Pten-KO (51)** | **<0.0001** |
| WT (48) vs. PtRap-DKO (23) | 0.9948 |
| WT (48) vs. PtRic-DKO (50) | 0.2072 |
| **Pten-KO (51) vs. PtRap-DKO (23)** | **0.0137** |
| **Pten-KO (51) vs. PtRic-DKO (50)** | **<0.0001** |
