# Supplementary material for: PTEN Regulates Microtubule Polymerization via mTORC2, But Not mTORC1, in Peripheral Sensory Neurons
Source: eNeuro. 2026 Sep 8;13(9):ENEURO.0116-26.2026. doi: 10.1523/ENEURO.0116-26.2026 (PMC13640863; doi:10.1523/ENEURO.0116-26.2026)
Supplement: Supplementary file 5 [file eneuro-13-ENEURO.0116-26.2026-s008.docx]

|  | Mean (sum of length, um) | Std. Dev. | n (cells) |
| --- | --- | --- | --- |
| Proximal WT | 570.6 | 370 | 48 |
| Proximal *Pten-*KO | 615.8 | 277.2 | 51 |
| Proximal *Pten-*KO x *Raptor-*KO | 870 | 495.1 | 23 |
| Proximal *Pten-*KO x *Rictor-*KO | 414.5 | 315.2 | 50 |
| Distal WT | 138.5 | 372 | 48 |
| Distal *Pten-*KO | 540.9 | 629.2 | 51 |
| Distal *Pten-*KO x *Raptor-*KO | 191.4 | 486.7 | 23 |
| Distal *Pten-*KO x *Rictor-*KO | 16.32 | 56.74 | 50 |
